# Activation of the VQ Motif-Containing Protein Gene *VQ28* Compromised Nonhost Resistance of *Arabidopsis thaliana* to *Phytophthora* Pathogens

**DOI:** 10.3390/plants11070858

**Published:** 2022-03-24

**Authors:** Xingjie Lan, Xiaoxia Wang, Quandan Tao, Haotian Zhang, Jinyang Li, Yuling Meng, Weixing Shan

**Affiliations:** 1 State Key Laboratory of Crop Stress Biology in Arid Areas, Northwest A&F University, Xianyang 712100, China; xjlan@nwafu.edu.cn (X.L.); wxiaoxia@nwafu.edu.cn (X.W.); qdtqo@nwafu.edu.cn (Q.T.); htzhang@nwafu.edu.cn (H.Z.); lijinyang66307@163.com (J.L.); mengyuling@nwafu.edu.cn (Y.M.); 2 College of Plant Protection, Northwest A&F University, Xianyang 712100, China; 3 College of Agronomy, Northwest A&F University, Xianyang 712100, China

**Keywords:** plant immunity, nonhost resistance, susceptibility, *VQ28*, *Phytophthora*

## Abstract

Nonhost resistance refers to resistance of a plant species to all genetic variants of a non-adapted pathogen. Such resistance has the potential to become broad-spectrum and durable crop disease resistance. We previously employed *Arabidopsis thaliana* and a forward genetics approach to identify plant mutants susceptible to the nonhost pathogen *Phytophthora sojae*, which resulted in identification of the T-DNA insertion mutant *esp1* (*enhanced susceptibility to Phytophthora*). In this study, we report the identification of VQ motif-containing protein 28 (*VQ28*), whose expression was highly up-regulated in the mutant *esp1*. Stable transgenic *A. thaliana* plants constitutively overexpressing *VQ28* compromised nonhost resistance (NHR) against *P. sojae* and *P. infestans*, and supported increased infection of *P. parasitica*. Transcriptomic analysis showed that overexpression of *VQ28* resulted in six differentially expressed genes (DEGs) that are involved in the response to abscisic acid (ABA). High performance liquid chromatography-mass spectrometry (HPLC-MS) detection showed that the contents of endogenous ABA, salicylic acid (SA), and jasmonate (JA) were enriched in *VQ28* overexpression lines. These findings suggest that overexpression of *VQ28* may lead to an imbalance in plant hormone homeostasis. Furthermore, transient overexpression of *VQ28* in *Nicotiana benthamiana* rendered plants more susceptible to *Phytophthora* pathogens. Deletion mutant analysis showed that the C-terminus and VQ-motif were essential for plant susceptibility. Taken together, our results suggest that *VQ28* negatively regulates plant NHR to *Phytophthora* pathogens.

## 1. Introduction

Most plants can suppress numerous diseases found in nature, which implies that susceptibility is more of an exception than the rule [1]. Two types of immune response against pathogen invasion have been defined: host and nonhost resistance. Gene-for-gene mediated host resistance is commonly used in crop breeding. Unfortunately, introducing *R* genes in crop cultivars results in drastic but non-durable conflicts with specific pathogens, since pathogens evolve rapidly and appearance of a new race has the potential to overcome the resistance. Alternatively, nonhost resistance (NHR) is recognized as the immunity of a plant species to most genetic variants of a non-adapted pathogen species. NHR mechanism studies have revealed a multilayer trait in which physical and chemical barriers function to prevent pathogen invasion. The cuticle layer, epidermis, and cell wall are natural physical barriers present in all plants that block pathogen access [2,3,4]. Some secondary metabolites, such as coumarin, scopoletin, and camalexin, are directly involved in restricting the growth of ingressing pathogens [5,6,7]. NHR is therefore considered to contribute broad and durable disease resistance in crop plants.

NHR is often triggered by pathogen-associated molecular patterns (PAMPs) through pattern recognition receptors (PRRs) on plant membranes. Forsyth et al. [8] found that the response to *P. syringae* pv. *phaseolicola* was affected by *FLS2* gene dosage. PEN3, which is required for *A. thaliana* NHR, contributes to defenses at the cell wall and intracellularly. *pen3* mutants make *A. thaliana* susceptible to various non-adapted pathogens such as powdery mildews, rust fungi, and oomycetes and also alter susceptibility to adapted bacterial pathogens [9,10,11]. Infections with *Fusarium virguliforme*, a necrotrophic fungal pathogen causing sudden death syndrome in soybean, and *P. sojae* were demonstrated in *pss*1 mutant [12,13]. When glycine-rich plasma membrane PSS1 protein is introduced into the soybean cultivar Williams 82 (W82) driven by Prom2 (a soybean root-specific promoter) and Ubi10 (an *Arabidopsis* constitutive promoter), the transgenic crops exhibit enhanced resistance to *F. virguliforme* [13]. Recently, the *A. thaliana PSS30* gene which encodes a folate transporter, AtFOLT1, was found to enhance broad-spectrum disease resistance in the soybean cultivar W82 against *F. virguliforme* and soybean cyst nematode [14]. If pathogens fail to invade nonhost plants due to the activation of basal host resistance triggered by PAMPs (PTI), they will release effectors to gain nutrition and interfere with host physiology, leading to the development of effector-triggered susceptibility (ETS). Host plants then express *R* genes encoding receptors that recognize the effectors and trigger immunity (ETI), leading to a hypersensitive response (HR) or programmed cell death [15]. Several defense responses induced during plant NHR are similar to those induced during host resistance.

The valine-glutamine (VQ) motif-containing proteins comprise a conserved core motif FxxhVQxhTG where h denotes hydrophobic residues and x represents any amino acid [16,17,18,19,20]. Most *A. thaliana VQ* genes do not have introns, and encode relatively small proteins with no more than 300 amino acids. Most are predicted to localize to the nucleus, while some localize to plastids, including mitochondria [21]. The *A. thaliana* VQ proteins are grouped into seven or ten clusters, according to phylogenetic analyses and structural features of the VQ domain or full-length sequences [22,23]. The VQ family members show huge differences in genome sequences except the VQ-motif. VQ proteins are detectable not only in plants but also in some fungi, lower animals, and bacteria, functioning in responses to abiotic and biotic stress or other biological processes through various mechanisms [17].

*JAV1* (*VQ22*), a key gene in the jasmonate (JA) pathway, functions in antagonistically regulating resistance to protect plants from insect attack and pathogen infection without a detectable role in plant development [24]. Additionally, the JAV1-JAZ8-WRKY51 (JJW) complex counteracts expression of JA biosynthesis genes in healthy plants and injury rapidly triggers Ca^2+^/calmodulin dependent phosphorylation of JAV1 to disrupt the JJW complex, thereby activating JA biosynthesis for plant defense [25]. JAV1 is ubiquitinated through interacting with JUL1 in the nucleus, which modulated the expression levels of the JA-marker gene *PDF1.2* and led to proteasome disintegration of JAV1, ultimately coordinating plant defense without disrupting plant development or growth [26]. Transgenic *A. thaliana* lines where the VQ-motif is mutated from LVQK residue into EDLE cannot rescue the phenotype of *wrky2-1 wrky34-1 vq20-1* triple mutant plants, confirming that the VQ-motif of VQ20 is critical for pollen development [27]. Many VQs from other plants have been identified as regulator of plant immunity. Zou and the colleagues revealed that overexpression of *BnVQ7* led to an increased resistance to *Leptosphaeria maculans* infection at the adult plant stage [28]. Liu et al. identified 59 VQs in *Nicotiana tabacum*, with expression of more than half *NtVQ* genes significantly induced upon *Ralstonia solanacearum* infection [29]. The poplar *VQ1* conferred various biotic and abiotic stress responses in transgenic *Arabidopsis* lines by mediating abscisic acid and salicylic acid [30]. Silencing soybean *GmVQ58*, which is responsive to *Spodoptera litura* infection, improved plant defense against *S. litura* via interaction with GmWRKY32 [31]. In *Cucurbita pepo*, 44 *VQ* genes were identified to be differentially regulated under some abiotic stresses and powdery mildew infection, indicating their important roles in plant stress responses [32]. León et al. summarized potential core regulatory roles of VQ proteins in NO-modulated and O_2_-modulated defense responses [33].

The genetic basis of NHR against pathogens is far from clear, especially for pathogenic oomycetes. *A. thaliana* is used to determine the NHR mechanism with *P. sojae* [12,34] and *P. infestans* [9,35,36], and it can also be infected by *P. parasitica*, a typical soil-borne pathogen that can infect plants from more than 255 genera in 90 families [37,38]. We employed the *A. thaliana*-*P. sojae* interaction to address NHR to oomycete pathogens. By screening a collection of *A. thaliana* mutants for enhanced susceptibility to *P. sojae*, we identified a T-DNA insertion mutant *esp1* and confirmed the occurrence of four T-DNA insertion events [34]. In this study, we show that the expression of *VQ28* (*AT4G20000*), which is localized upstream of one T-DNA insertion site and belongs to the VQ-motif family, is highly activated in *esp1* and is responsible for the loss of NHR to *P. sojae* and *P. infestans* and for enhanced susceptibility to *P. parasitica*. Furthermore, we show that the C-terminal residues and VQ-motif function directly in plant susceptibility to *Phytophthora*.

## 2. Results

### 2.1. VQ28 Expression Was Highly Activated in the T-DNA Insertion Mutant esp1

**Figure 1:**
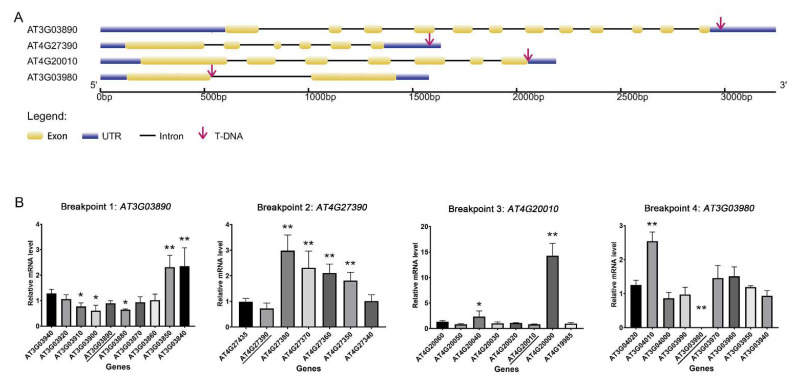
Identification of the insertion loci and the expression values of genes in the vicinity of the T-DNA. (**A**) Schematic illustration of the locus surrounding T-DNA insertion site in *esp1* mutant. (**B**) The expression of the genes in the vicinity of the T-DNAs in *esp1* leaves without pathogen infection. Total RNA of wild-type and *esp1* was isolated from leaf samples, and transcript levels were calculated using qRT-PCR with *A. thaliana EF1A* gene as internal control. The data points in Figure 1B represented mean expression values of genes in *esp1* mutant, compared with WT Col-0. Since the distinct means from both *esp1* and Col-0, we used Student’s *t*-test to compare the two sets of every gene’s expression levels. Error bars indicate standard errors (*n* = 3). Asterisks indicate significant differences between expression values of *esp1* and Col-0 (** *p* < 0.01, * *p* < 0.05).

Southern blot analysis has indicated that at least four T-DNA insertion sites are involved in *esp1* and further genetic analysis revealed that susceptibility to infection in the mutant was likely controlled by a single recessive locus [34]. We thus employed whole-genome sequencing (WGS) to find insertion events specific to *esp1*. Sequence alignment showed the exact insertion sites revealing at least four T-DNA insertion breakpoints (*AT3G03890*, *AT3G03980*, *AT4G20010*, and *AT4G27390*) in the mutant (Figure 1A). As showed in Figure 1A, breakpoints 1 and 2 were respectively detected in the 3′-untranslated region (3′-UTR) of *AT3G03890* and *AT4G27390*. Breakpoint 3 was detected in the last exon of gene *AT4G20010* just next to the stop codon. These three insertional events were hardly decreased their mRNA levels which was confirmed by the followed quantitative RT–PCR analysis (Figure 1B). Meanwhile, breakpoint 4 occurred in the first open reading frame of *AT3G03980*. Following verification, we explored the mRNA levels of genes in the vicinity of the T-DNAs by quantitative RT-PCR analysis (Figure 1B). This showed that *AT3G03980* in *esp1* was completely knocked out. Transcript levels of *AT3G03890*, *AT4G20010*, and *AT4G27390* were slightly lower compared to WT. More interestingly, the expression of *VQ28* (*AT4G20000*) was up-regulated by more than 15-fold whereas expression levels of other genes flanking all the insertion sites were low.

### 2.2. Overexpression of VQ28 Significantly Compromised Nonhost Resistance to P. sojae and P. infestans

**Figure 2:**
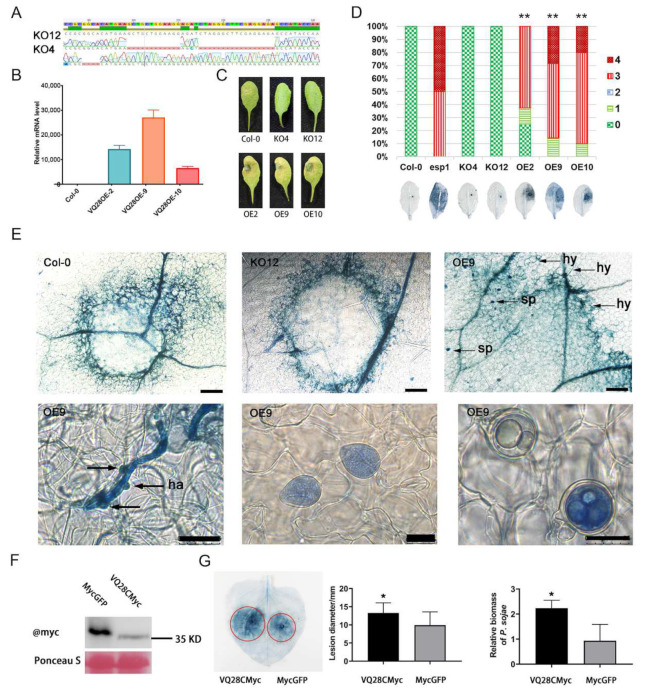
*VQ28* controlled plant defense against *P. sojae*. (**A**) Mutation locations and forms by direct sequencing of PCR products containing targeted sites in *Arabidopsis* T3 plants. (**B**) Transcript levels of the *VQ28* gene significantly increased in overexpression lines, as detected by qRT-PCR assays with *A. thaliana EF1A* gene as an internal control. (**C**) The phenotypes of WT, two cas9 knock-out lines (KO4 and KO12), and three *VQ28* overexpression plants (OE2, OE9, and OE10) at 60 h after inoculation with *P. sojae*. (**D**) Graph of ratio for susceptibility levels of the plants. Columns with double asterisks (** *p* < 0.01) were statistically different compared to the wild type Col-0 according to Wilcoxon’s Signed Rank Test. Trypan blue-stained leaves of the plants below indicated in (**C**). (**E**) Photomicrographs of *A. thaliana* wild-type Col-0, *VQ28* knock-out mutant KO12, and overexpression line OE9 trypan blue stained leaves inoculated by *P. sojae*. Arrows indicate the hyphae (hy), haustoria (ha), sporangia (sp), or oospore (oo). Bars, 200 μM in the first line and 25 μM in second line. (**F**) The protein was extracted from *Nicotiana* *benthamiana* leaves infiltrated with *Agrobacterium tumefaciens* GV3101 with 35S::VQ28CMyc and 35S::MycGFP and a western blot showed the correct protein band. (**G**) *N. benthamiana* leaves expressing VQ28CMyc and MycGFP constructs were challenged by *P. sojae*. At least 8 leaves per biological replicate were used in three independent experiments. The statistical analysis by Student’s *t*-test of lesion diameters indicated that overexpression of VQ28 enhanced the infection of *P. sojae*. *P. sojae* colonization in the infected plant tissues at 3 dpi was determined by qRT-PCR. Primers specific for *P. sojae UBC* gene and *N. benthamiana NbActin* were used. Bars represent standard errors from three biological replicates and asterisks are statistical significance based on Student’s *t*-test (** *p* < 0.01, * *p* < 0.05).

Previous study showed that *AT3G03890*, *AT4G20010*, and *AT4G27390* which were inserted by a T-DNA fragment were irrelevant to the susceptibility of *esp1* [39,40]. The specifically induced expression of *VQ28* caused by the single adjacent T-DNA insertion in *AT4G20010* was investigated in more detail. *VQ28* encodes a VQ motif-containing protein with 208 amino acids. To further confirm its role in plant immunity, we created *VQ28*-knockout mutants using CRISPR/Cas9 method and prepared *VQ28*-overexpression transformants. Two individual homozygous lines KO12 and KO4 were chose to perform infection assays. Mutant KO12 contains two deletions with 11-bp and 17-bp fragments and mutant KO4 contains a 4-bp deletion; all deletions were in the coding region of *VQ28* (Figure 2A). The deletions led to a frameshift mutation and truncated protein. The qRT-PCR data showed that the *VQ28* transcript levels were significantly increased in three overexpression lines (OE2, OE9, and OE10) (Figure 2B). The homozygous Col-0 background cas9 knock-out T3 plants and overexpression lines without altered developmental phenotype were chosen for pathogen infection analysis.

At 3 days post infection (dpi) with *P. sojae*, the inoculated leaves of OE2, OE9, and OE10 showed more serious water-soaked lesions, and increased disease severity and lesion sizes. The knock-out mutants KO4 and KO12 showed NHR like Col-0 (Figure 2C–E). To confirm if *P. sojae* became adapted to *A. thaliana* overexpressing *VQ28*, a microscopic study of the disease lesions on the detached leaves was carried out at different time points. Detached leaves of KO12 and wild-type (WT) Col-0 were rarely infected by *P. sojae*. Nevertheless, we observed enhanced hyphal growth and formation of haustoria (24 h post infection (hpi)), sporangia, and oospores (5 dpi) in VQ28OE9 leaves (Figure 2E). Additionally, a heavy colonization was evident in VQ28OE9 when infected with Pi14-3, a *P. infestans* transformant constitutively expressing GFP (Figure S1). The susceptibility was caused by the constitutive higher mRNA level of *VQ28*, a negative regulator that functions in plant immunity against *Phytophthora*. We confirmed this by infiltrating *Agrobacterium* carrying 35S::VQ28CMyc in *N. benthamiana* leaves and inoculating *P. sojae* two days later. Western blot analysis confirmed protein integrity (Figure 2F). Significantly, less *P. sojae* colonization was perceived in control tobacco leaves, showing that *VQ28* accelerated *P. sojae* invasion (Figure 2G).

We performed the same infection assay with *P. infestans* and *P.* *parasitica*. *VQ28* overexpressing *A. thaliana* consistently developed more severe disease symptom than in the WT *A. thaliana* at 60 hpi (against *P. parasitica*) or 7 dpi (against *P. infestans*) (Figure S2A,B). The ratio of susceptible leaves was much higher in *VQ28* overexpression lines than in Col-0 (Figure S2C,D). In contrast to *VQ28* overexpression plants, there were no macroscopic disease symptoms on WT Col-0 and *VQ28* knock-out lines after infection with incompatible *P. sojae* (Figure 2C,D) and *P. infestans* (Figure S2A,C). Indistinct disease severity and pathogen colonization was exhibited when challenged with compatible *P. parasitica* (Figure S2B,D). There was a significant increase (three- to five-fold) in pathogen mycelial colonization in 35S::*VQ28* transgenic plants compared with Col-0 at 48 hpi (Figure S2E). Consistently, the constitutive overexpression of *VQ28* led to increased growth of the pathogen and enhanced development of disease symptoms in *N. benthamiana* leaves (Figure S2F,G).

As it was knocked out in the susceptible mutant *esp1*, we further examined if *AT3G03980* had a positive role in plant immunity. Quantitative RT-PCR analysis of the gene profile after *Phytophthora* inoculation suggested that *AT3G03980* was barely induced (Figure S3). Thus, we generated transgenic lines that constitutively overexpressed *AT3G03980* under control of the CaMV 35S promoter, and generated RNA silenced transgenic plants in the WT Col-0 background. Three homozygous overexpression T3 lines (980OE2, 980OE3, and 980OE5) and knock-down lines (980KD6, 980KD9, and 980KD16) were confirmed by qRT-PCR (Figure S4A). When challenged with *P. parasitica*, the WT Col-0 detached leaves showed typical water-soaked lesions at 2 dpi. However, the loss-of-function lines did not mimic the susceptible phenotype of the *esp1* mutant, while the results suggested that *AT3G03980* does not play a role in plant immunity (Figure S4B,C).

### 2.3. VQ28 Was Responsive to Phytophthora Infection and Wounding

To visualize the expression pattern of *VQ28*, we generated transgenic lines expressing the β-glucuronidase reporter gene under the control of its native promoter sequence (about 2.5 kb upstream of the ATG translational start codon). Histochemical staining showed that the GUS reporter gene was expressed in the embryo, radicle, stem, leaf, and flower, suggesting that *VQ28* may function in these tissues (Figure S5).

**Figure 3:**
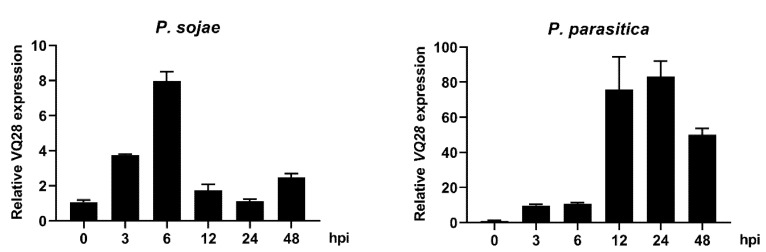
Induced gene expression level of *VQ28* by Col-0 leaves upon *P. sojae* and *P. parasitica* infection. The qRT-PCR data showed *VQ28* was up-regulated during infection. Total RNA of wild-type Col-0 was isolated from leaf samples, and transcript levels were detected by qRT-PCR with *A. thaliana EF1A* gene as an internal control. Error bars indicate SD (*n* = 3).

To determine if *VQ28* was pathogen responsive, we further analyzed the induced expression in response to *P. sojae* and *P. parasitica* infections. Results showed that *VQ28* expression was induced by both pathogens during the infection. Starting at 3 h post infection of *P. sojae*, the expression level showed a stably increase, and then a peak come out at 6 hpi before a decrease at 12 hpi. Meaning while, the mRNA level sharply rose from 3 hpi to a peak at 24 hpi, followed by a drop at 48 hpi after *P. parasitica* infection (Figure 3). Using our transcriptome data generated by RNA-seq in roots [41] and leaves [42] of Col-0 infected *P. parasitica*, we examined the biological function of *VQ* genes by analyzing transcriptional changes of the gene family during *P. parasitica* infection. In the infected leaves (Figure S6A), *VQ28* and three other *VQ* genes (*VQ13*, *VQ27*, and *VQ29*) were induced, at about a three-fold increase, while *VQ8* and *VQ17* were highly expressed at 24 hpi. *VQ28* was correspondingly induced up to a four-fold increase in the infected roots (Figure S6B). We also showed that *VQ28* transcription was induced by wounding (Figure S7).

### 2.4. Overexpression of VQ28 Was Involved in ABA-, SA-, and JA-Signaling Pathways

**Figure 4:**
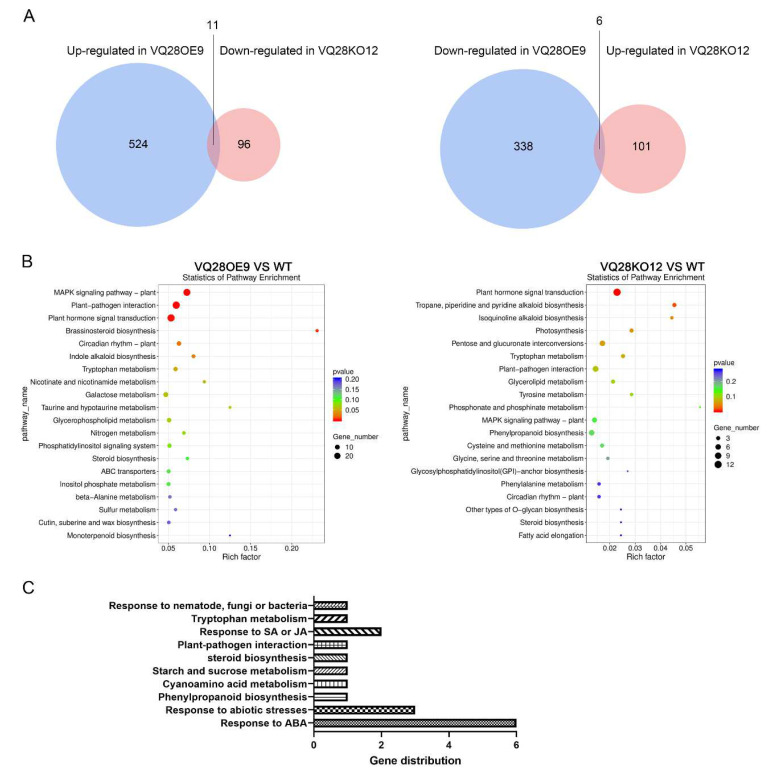
Global transcriptional changes in uninfected wild-type Col-0 (WT), knock-out plant VQ28KO12, and overexpression line VQ28OE9. (**A**) Number of genes in *A. thaliana* uninfected leaves. The numbers of differentially expressed genes (DEGs) showed the overlap of genes up-regulated in VQ28OE9 and down-regulated in VQ28KO12 compared with WT (left), and the overlap of genes down-regulated in VQ28OE9 and up-regulated in VQ28KO12 compared with WT (right). (**B**) KEGG pathway map analysis showed DEGs, mainly in MAPK signaling pathway and plant hormone signal transduction. The sizes and colors of the dots indicate the numbers of DEGs and the q-value, respectively. (**C**) The distribution showed the 11 overlapping genes up-regulated in VQ28OE9 but down-regulated in VQ28KO12; six of them responded to abscisic acid.

A transcriptome project was carried out using uninfected rosette leaves of Col-0, knock-out mutant VQ28KO12, and the overexpression transformant VQ28OE9. Compared to Col-0, there were 524 genes up-regulated and 338 genes down-regulated in VQ28OE9, including *VQ28* itself, whereas 101 genes were up-regulated and 96 genes were down-regulated in untreated VQ28KO12 (Figure 4A). Pathway enrichment analyses of the differentially expressed genes (DEGs) based on the Kyoto Encyclopedia of Genes and Genomes (KEGG) database were performed to study certain roles in the biological functions (Figure 4B). In comparison with Col-0, constitutively overexpressing *VQ28* caused enriched expression of genes in the mitogen-activated protein kinase (MAPK) signaling pathway and plant hormone signal transduction. Among the 11 genes up-regulated in VQ28OE9 but down-regulated in VQ28KO12 (Figure 4A), six were involved in the response to abscisic acid (ABA) (Figure 4C), according to their expression by querying the *A*. *thaliana* eFP Browser (http://bar.utoronto.ca/ (10 May 2021)) [43], which contains an extensive collection of gene expression microarray data. qRT-PCR analyses of expression consistently showed that five of six ABA-related genes (*AT1G69480*, *AT1G52690*, *AT3G02480*, *AT5G24110*, and *AT1G76930*) were significantly up-regulated in VQ28OE9 plants without infection (Figure S8). The gene *AT1G52690* was hardly detected in the wild-type Col-0 while it was significantly induced in VQ28OE9.

**Figure 5:**
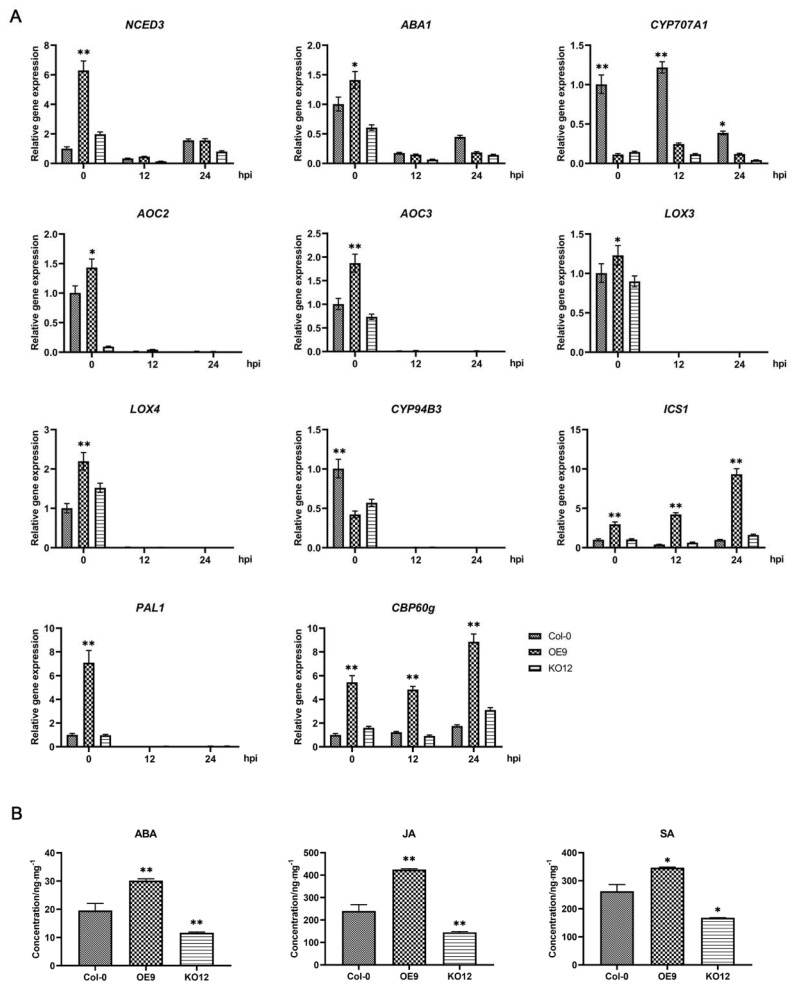
Overexpression of *VQ28* disturbed abscisic acid (ABA), jasmonic acid (JA), and salicylic acid (SA) signaling pathways. (**A**) Defense marker gene expression in the *A. thaliana* Col-0, knock-out line VQ28KO12, and overexpression line VQ28OE9. Total plant RNA was obtained from detached leaves infected with *P. sojae* at 0, 12, and 24 hpi. *A. thaliana EF1A* was used as the internal control and transcript levels relative to the wild-type Col-0 are shown. Bars represent SD from three biological replicates and asterisks indicate statistical significance based on a Student’s *t*-test (* *p* < 0.05, ** *p* < 0.01). Error bars indicate SD (*n* = 3). (**B**) The endogenous plant hormones in uninfected leaves of Col-0, knock-out plant VQ28KO12, and overexpression line VQ28OE9 were detected by HPLC-MS. Error bars indicate SD (*n* = 5).

As *VQ28* negatively mediated plant resistance to *Phytophthora* and was involved in ABA signal transduction, we measured hormone content and the expression of associated marker genes in Col-0, the overexpression (VQ28OE9) and knock-out (VQ28KO12) plants. We measured the mRNA levels of ABA, salicylic acid (SA), and JA signaling pathway related genes within 24 hpi in *P. sojae* infected plants. Overexpression of *VQ28* significantly induced the ABA biosynthesis genes *NCED3* and *ABA1* and decreased the expression of the catabolism gene *CYP707A1* without infection (Figure 5A). NCED3 is a rate-limiting enzyme that regulates ABA biosynthesis and ABA1 is involved in ABA biosynthesis [44]. *CYP707As*, encoding ABA 8′-hydroxylases, are key enzymes in ABA catabolism [45]. Nevertheless, the expression of *CYP707A1* showed continuous suppression during the infection. *AOC2* and *AOC3* encode allene oxide cyclase and are involved in JA synthesis [46]; both were upregulated in OE9 plants but downregulated in KO12 plants (Figure 5A). JA catabolic gene *CYP94B3* was downregulated in OE9 plants. *LOX3* and *LOX4* encode lipoxygenases [47] and were upregulated in the overexpression transformant OE9 at 0 hpi, but hardly detected during infection (Figure 5A). Moreover, the transcription level showed that *PAL1* (phenylalanine ammonia lyase 1), *ICS1* (isochorismate synthase), and *CBP60g* (cam-binding protein 60-like G), which are essential in SA synthesis [48,49], were significantly upregulated in the overexpression leaves (Figure 5A). The transcription of both *ICS1* and *CBP60g* was dramatically induced by *P. sojae* infection, especially during the early infection stage.

Endogenous ABA, JA, and SA in the uninfected 30-day-old rosette leaves of Col-0, knock-out line VQ28KO12, and overexpression plant VQ28OE9 were quantitated using high performance liquid chromatography-mass spectrometry (HPLC-MS) (Figure 5B). The results showed that the quantities of ABA, SA, and JA in VQ28OE9 were continually enriched much more than in Col-0 leaves. These results indicated that *VQ28* participated in ABA, SA, and JA signaling pathways.

### 2.5. The C-Terminus and VQ-Motif Functioned Directly in Promoting Susceptibility to Phytophthora Pathogens

**Figure 6:**
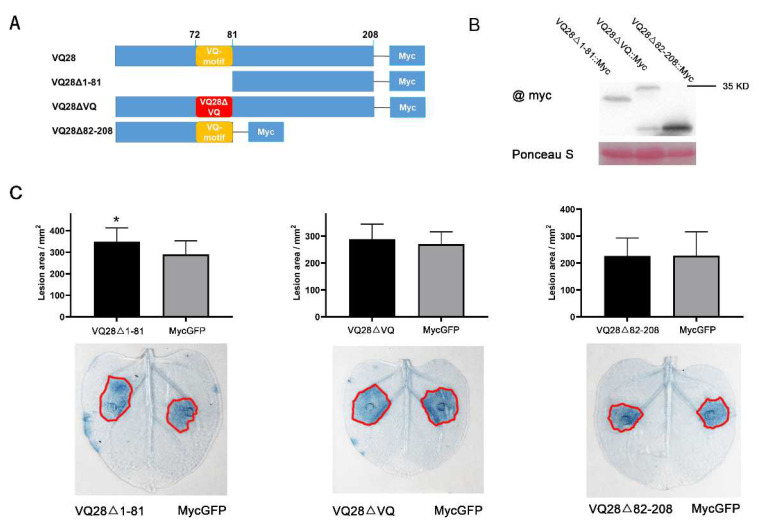
The C-terminal regions and VQ-motif of VQ28 are essential in susceptibility upon *P. sojae* infection. (**A**) Diagram of the full-length and truncated VQ28 constructs with specific deletions or mutations. (**B**) The protein was extracted from *N. benthamiana* leaves expressing VQ28Δ1-81::Myc (26.2 kDa), VQ28ΔVQ::Myc (34.8 kDa), and VQ28Δ82-208::Myc (20.4 kDa) and a western blot showed the correct protein band. (**C**) Quantification of lesions in *N. benthamiana* leaves measured at 60 hpi. Error bars represent the standard deviation (SD) of more than eight leaves, and asterisks denote significant differences from the MycGFP control group (Student’s *t*-test; * *p* < 0.05).

To identify which domain was responsible for the susceptibility function of *VQ28*, we designed mutants to successively delete the NH_3_-terminus (VQ28Δ1-81) and COOH-terminus (VQ28Δ82-208), and mutate the VQ-motif (VQ28ΔVQ). We constructed three Myc-tagged mutants and transiently expressed them in *N. benthamiana* leaves (Figure 6A). Agar disks containing *P. sojae* mycelium were inoculated on both sides of each leaf expressing *VQ28* structure mutants and MycGFP. Western blot analysis confirmed integrity of the mutant proteins (Figure 6B). Lesion diameters were measured at around 60 hpi. Compared with leaves expressing MycGFP, N-terminal deletion of *VQ28* promoted *P. sojae* infection. However, replacing LVQ in the VQ-motif (75–77 aa) with EDL or deleting the C-terminal residues eliminated its susceptibility function (Figure 6C). In addition, inoculations with *P. parasitica* and *P. infestans* on *N. benthamiana* leaves transiently over expressing VQ28Δ1-81::Myc also rendered a susceptible phenotype (Figure S9).

## 3. Discussion

Oomycete pathogens cause serious diseases in many commercially important crops. The genetic basis of NHR provides breeders an alternative for broad-spectrum and durable resistance to cope with gene-for-gene resistance that is rapidly overcome in plants. *VQ28* was identified as a negative immune regulator in this study, which compromised NHR, enhancing plant susceptibility to *P. sojae* and *P. infestans* in *A. thaliana* and *N. benthamiana*, and overexpression of *VQ28* showed increased susceptibility to *P. parasitica*. C-terminal regions and VQ-motif were demonstrated to have critical roles in plant immunity. *VQ28* was shown to partially participate in signaling pathways of ABA, JA, and SA. Overexpression of *VQ28* increased the contents of ABA, JA, and SA with supposed plant cell damages by excessive ROS burst, which remained to be studied in detail.

T-DNA insertion mutagenesis is a convenient method to explore new genes and their functions. In our lab, several negative regulators of certain pathogen defenses were identified from *Arabidopsis* T-DNA insertion mutants in the compatible *Arabidopsis*-*P. parasitica* pathosystem [50,51,52,53]. Initially, four genes disturbed by T-DNA insertion in *esp1* were confirmed by thermal asymmetric interlaced PCR (TAIL-PCR) and southern blot [34]. Next-generation sequencing based methods have been utilized to identify insertions of exogenous fragments in *A. thaliana* [54,55], rice [56,57], and maize [58]. WGS was therefore used to confirm four T-DNA insertion loci in chromosomes 3 (*AT3G03890* and *AT3G03980*) and 4 (*AT4G20010* and *AT4G27390*) of *esp1*. A new insertion locus in gene *AT3G03980* was identified and confirmed as knocked out by real-time RT-PCR (Figure 1).

Considering transgenic events with sophisticated modifications, rearrangement/deletion of exogenous fragments, or individual nucleotide substitutions, TAIL-PCR performed in *esp1* is not enough to identify all insertion sites [56,59]. Consistently, individual mutants homozygous for *AT4G27390*, *AT4G20010* and *AT3G03890*, respectively showed similar responses as that of wide type Col-0 to the infection by *P*. *sojae* [39,40]. However, genetic research demonstrated that *AT3G03980* knockout or overexpression lines showed no difference in defense response to *Phytophthora* invasion (Figure S4). Genetic analysis suggested that the susceptibility of *esp1* come from genetic variation in chromosome 4 [39]. We checked the biological process of genes in chromosome 4 with significant expression levels (*AT4G27380*, *AT4G27370*, *AT4G27360*, *AT4G27350*, *AT4G20040*, and *AT4G20000*) between *esp1* and Col-0 on the Arabidopsis Information Resource (TAIR, www.arabidopsis.org (10 May 2021)). All the six genes are not relevant with plant resistance, exclude the hyper-activated gene, *AT4G20000* (*VQ28*), which takes part in defense response to fugus, bacterium and so on. Therefore, we focused on *VQ28* to explore the plant nonhost resistance to *Phytophthora*. Three *VQ28* overexpression lines and *N. benthamiana* leaves expressing a VQ28CMyc fusion protein elevated the pathogen colonization levels of *P. sojae*, *P. infestans*, and *P. parasitica* (Figure 2, Figures S1 and S2). Unfortunately, two cas9 mutants destroying the *VQ28* amino acid sequence did not display improved resistance phenotypes after inoculating *Phytophthora* spp (Figure 2 and Figure S2). The important hallmarks of a successful adapted pathogen are its ability to launch feeding structures, obtain nutrition from the host, and finally complete its lifecycle in the infected plant [60]. Observation of trypan blue staining showed that incompatible *P. sojae* penetrated the leaf surface successfully in VQ28OE9 based on attacked epidermal cells and formed haustoria, sporangia, or oospores (Figure 2E). Thus, *VQ28* was identified as a negative immune regulator resulting in the susceptible phenotype of *esp1* infected by *P. sojae*. While up-regulated expression of *VQ28* led to plant susceptibility to non-adapted *Phytophthora* pathogens, its knock-out transformants did not show enhanced resistance. We are not sure the exact underlying mechanisms *VQ28* plays in plant immunity, it’s possibly relevant to fine-tuning of ROS levels. *VQ28*, a protein functioning in nuclear, has potential complicated interactions with transcription factors such as well-documented WRKYs, and further investigation will facilitate improved understanding of VQ28-mediated plant immune regulation.

T-DNA integration in plant genomes usually results in knockdown or knockout of a gene. In our study, four insertion events were identified in *esp1*, but only one resulted in knockout of *AT3G03980*. Genetic evidence highlighted that *AT3G03980* was unrelated to the susceptibility of *esp1* (Figure S4). Nevertheless, these data demonstrate that *VQ28*, which was induced based on a high mRNA level caused by the T-DNA insertion, functioned directly in plant susceptibility and showed decreased plant NHR and host resistance. In addition to the microscopic examination upon invasion, overexpressing *VQ28* not only abolished NHR to *P. sojae* and *P. infestans*, but also impaired host resistance to *P. parasitica* (Figure 2, Figures S1 and S2). We thus speculated that *VQ28* identified here negatively mediated the compromised NHR and host resistance. Occasionally, a single gene variant could compromise plant NHR and switch an incompatible interaction into compatibility. The deduced *VQ28* identified through mutations compromised the NHR against *P. sojae*, resulting in a compatible interaction with *Arabidopsis*, and demonstrating a negative role of *VQ28* in both host and nonhost immunity against oomycetes. Recent studies indicate that the molecular basis of NHR is highly complex and might overlap with host resistance. Researchers proposed that *Arabidopsis* heterotrimeric G-proteins and small GTPase NOG1 with GTPase activity contributed to both host resistance and NHR [61,62]. GENERAL CONTROL NONREPRESSIBLE4 (GCN4), an AAA^+^-ATPase family protein, mediates host and nonhost disease resistance due to open stomata during pathogen infection in *N. benthamiana* and *A. thaliana* [63]. The isolate-specific avirulence effector AvrPm3 interacts with a nucleotide binding leucine rich repeat (NLR) immune receptor Pm3 revealing a unique model system for understanding how NLRs can contribute to both host and nonhost resistance [64].

Expression analysis showed that *VQ28* was up-regulated upon *Phytophthora* infection and wounding (Figure 3, Figures S6 and S7). The transcript levels of *Arabidopsis VQ*s are induced or repressed by pathogen invasion [21,24,26], highlighting the underlying capacity of *VQ* genes in disease management. The *VQ12* and *VQ29* genes are hyper-induced by *Botrytis cinerea*. Two *amiR*-mediated transgenic lines showed reduced disease while two overexpression lines accumulated more fungal colonization, showing that both VQ proteins negatively mediate plant basal resistance against necrotrophic fungal pathogen *B. cinerea* [65]. Results obtained by Chen et al. [66] revealed that *VQ10* expression was hugely responsive to *B. cinerea* and defense-related hormones and destruction of *VQ10* enhanced mutants’ susceptibility against *B. cinerea,* whereas constitutively-expressing *VQ10* enhanced resistance.

According to the RNA-seq data, overexpression of *VQ28* caused the enrichment of genes involved in MAPK and plant hormone signaling pathways (Figure 4B). VQs interact with WRKY transcription factors (WRKYs) to form a compound and regulate plant immunity [25,67,68,69]. Many WRKYs regulate pathogen resistance via different plant hormones and some that interacted with VQs were involved in the MAPK signaling pathway. In this study, we found that 14 *WRKYs* were enriched in VQ28OE9 (Table S2). We further analyzed the expression patterns of co-expressed *WRKY* genes with *VQ28* in leaves infected with *P. parasitica*, and revealed five *WRKY* genes (*WRKY6*, *WRKY8*, *WRKY17*, *WRKY30*, and *WRKY47*) showed the same while *WRKY33* displayed opposite expression profiles as compared with *VQ28* (Figure S10). These results suggested that these *WRKY* genes might cooperate with *VQ28* to function in plant susceptibility upon *Phytophthora* invasion. As shown by the similar or opposite expression levels, we speculate that these *WRKY* genes were probably involved with *VQ28* in plant resistance to *Phytophthora*.

We found that six differentially expressed genes (DEGs) up-regulated in VQ28OE9 but down-regulated in knockout VQ28KO12 were associated with ABA signaling (Figure 4C). Further qRT-PCR analysis demonstrated that overexpressing *VQ28* led to enrichment of ABA-, SA-, and JA-mediated signaling pathways due to suppression of catabolic genes and induction of biosynthesis genes (Figure 5A). Continuous detection of plant hormones revealed that ABA, JA, and SA were significantly induced in overexpression lines compared with WT Col-0 (Figure 5B). It seems to be that ABA show an antagonism or synergism with SA and JA in different plant-microbe interaction [70]. However, the evidence so far cannot declare a definite signaling pathway which *VQ28* play a key role in it. Interesting, it is notable that all three plant endogenous hormones (ABA, JA and SA) are senescence-promoting factors [71]. ROS are actively generated during senescence, which, in turn, lead to permanent alterations in redox regulating components [72]. ROS are negative to plant cells when excessively produced or when the antioxidant system is not efficient enough to scavenge them [73]. Therefore, we speculate that the increased contents of ABA, JA and SA may have broken hormonal balance, leading to enhanced leaf senescence and plant susceptibility to *Phytophthora*. The supposed leaf senescence probably functions in VQ28-mediated susceptibility, which attracted our interest and will be part of future research.

The secondary structure of VQ proteins affects their biological function. Multiple studies show that VQ-motif or C-/N-terminal residues function in plant resistance, growth, and development and interactions with other kinases or transcription factors [25,74,75,76,77]. In this study, three mutated VQ proteins with Myc tagged fusion, deleted NH_3_-terminal (VQ28Δ1-81), COOH-terminal (VQ28Δ82-208), and point mutated VQ-motif (VQ28ΔVQ) were transiently expressed in *N. benthamiana*. After *Phytophthora* inoculation, the previously elevated susceptibility disappeared in the leaves expressing VQ28Δ82-208 and VQ28ΔVQ, which confirmed the indispensable function of the C-terminus and VQ-motif in pathogen defense (Figure 6 and Figure S9). VQ-motif or the C/N-terminus participate in plant-microbe interactions. Phenotypic results showed that VQ12 and VQ29 interacted with themselves or each other via their C-terminal ends, and interacted with WRKY33 via the VQ-motif to form a big protein complex to mediate plant defense responses against *B. cinerea* [65]. Chen et al. [66] found that the mutant VQ10 with the site-mutated VQ-motif, the amino acids (LVQR) mutated to EDLE, failed to interact with WRKY8, suggesting an important contribution of the VQ-motif to the VQ10-WRKY8 interaction. Although deletion of N-terminal ends containing VQ-motif weakened the interaction between both VQ14-MINI3 and VQ21-WRKY33, the function of the VQ-motif was unclear in this interaction [78,79].

In conclusion, we confirmed that the susceptibility of T-DNA insertion mutant *esp1* was mediated by *VQ28*, which was particularly induced by a T-DNA integration. *VQ28* negatively regulated plant host and nonhost resistance to *Phytophthora* pathogens. Future research will be directed to understanding which WRKYs interact with *VQ28* and how this interaction triggers susceptibility upon *Phytophthora* invasion. It’s also interesting to explore whether *VQ28* functions as a negative immune regulator to the infection by other pathogens.

## 4. Materials and Methods

### 4.1. Plant Materials and Growth Conditions

Plants were grown in a growth chamber under normal conditions (13 h light/11 h dark cycle and 23 ± 2 °C temperature). For screening homozygous transformants, *Arabidopsis* seeds were surface-sterilized with 1% NaClO for 10 min and cleaned with sterilized water before being sown on half-strength Murashige and Skoog (1/2 MS) medium with kanamycin (pART27 vector backbone [80]) or hygromycin (pMDC162 and pCXSN vector backbone [81,82]). Then, 10-day-old seedlings were transferred to soil for infection assays. The cas9, RNA silenced, and overexpression lines in the *A. thaliana* ecotype Columbia-0 (Col-0) background were obtained using the *Agrobacterium tumefaciens*-mediated floral dip transformation method [83,84]. The RNA silenced and overexpression transformants were screened on 1/2 MS medium with kanamycin and were identified by expression levels using qRT-PCR. Gene editing was conducted as described previously [85] and the homozygous mutants were isolated from T2 generation plants by sequencing. Two or three independent T3 homozygous transformants were used in subsequent experiments. *N. benthamiana* plants were routinely cultured in soil in a chamber for about 5–6 weeks under the same conditions used for the growth of *A. thaliana*.

### 4.2. Vector Construction

All the primers and vectors used in this study are listed in Table S1. qPrimerDB [86] was used to design most of the gene-specific qPCR primers. Gene fragments were amplified and digested using EasyTaq^®^ DNA Polymerase (Transgen, Beijing, China) and appropriate restriction endonucleases (Thermo Scientific, Waltham, MA, USA) specified below. To obtain VQ28-cas9 vector, two sgRNAs, both targeting *VQ28*, were introduced into the pAtU6-sgRNA- pAtUBQ-Cas9-tUBQ vector backbone. Finally, the resulting construct was digested by *EcoR*I and *Hind*III and then inserted into the pCXSN vector for *Arabidopsis* transformation. The overexpression plasmid was obtained by cloning the full-length CDS of *VQ28* into pART27 behind the CaMV 35S promoter. For GUS reporter analysis, the native promoter of *VQ28* (2480 bp) was amplified from genomic DNA and the pMDC162 vector was digested with *Sgs*I and *Kpn*I. The resulting construct contained the pMDC162 vector backbone with GUS driven by the *VQ28* native promoter. To introduce some mutations into *VQ28*, three different fragments fused with Myc, a deletion of *VQ28* N-terminal (VQ28Δ1-81), a mutation of VQ-motif (VQ28ΔVQ), and a deletion of *VQ28* C-terminal (VQ28Δ82-208), were amplified and In-Fusion cloned into *Xba*I and *Xho*I digested *VQ28* overexpression plasmid with BM seamless cloning kit (Biomed, Beijing, China).

### 4.3. Identification of T-DNA Insertion Sites

Whole genome sequencing (WGS) was performed to identify the T-DNA insertion events in *esp1* mutant. Genomic DNA was collected from 30-day-old *Arabidopsis* rosette leaves using the cetyltrimethylammonium bromide (CTAB) method. About 5 mg of genomic DNA was sheared to fragments with an average length of 400 bp to construct libraries with the Nextera DNA Sample Preparation Kit (Illumina, San Diego, CA, USA). The libraries were sequenced with the Illumina Hiseq4000 platform and 125-bp paired-end reads were generated. Sequencing and analysis were accomplished by Biomarker Technologies Corporation (Beijing, China). We confirmed the T-DNA insertion by PCR using a combination of a T-DNA border primer and a Col-0 gene-specific primer. The transcript levels of 41 genes in the vicinity of every T-DNA occurring in the *esp1* mutant were further confirmed by qRT-PCR.

### 4.4. Pathogen Infection Analyses

*Phytophthora sojae* strain P6497, *P. parasitica* strain GFP-tagged Pp016, and *P. infestans* strain GFP-tagged Pi14-3 were used for plant infection assays in our study. *P. sojae* and *P. parasitica* were cultured on 5% (*v*/*v*) CA (cleared carrot juice and agar) medium with 0.005% (*w*/*v*) β-sitosterol and 0.01% (*w*/*v*) CaCO_3_. *P. infestans* was grown on rye sucrose agar (RSA) medium at 16 °C for about 10 days before zoospore production. *P. sojae* [87], *P. parasitica* [88], and *P. infestans* [89] zoospores were produced as previously described.

For inoculation of *Arabidopsis* with *P. sojae*, every detached leaf was slightly wounded by a toothpick on one side and inoculated with mycelial plugs (5-day-old, 5 mm diameter) or zoospores (2 × 10^5^/mL, 10 μL droplets). Then, these leaves were placed in a plastic tray, and maintained at 23 °C and 100% relative humidity in the darkness to ensure infection. By 60 hpi, the infected leaves were observed under a microscope, stained by trypan blue [90], or analyzed to determine susceptibility ranks. All the infected leaves were divided into five grades depending on the disease symptoms and water-soaked area ratio: Grade 0, no symptoms; Grade 1, etiolated mesophyll area is no more than 50% without water-soaked symptom; Grade 2, etiolated mesophyll area is more than 50% without water-soaked symptom; Grade 3, water-soaked area is no more than half the leaf; Grade 4, water-soaked area is more than half the leaf. More than ten leaves from corresponding plants were tested in different experiments and at least three independent experiments were conducted. Unlike inoculating *P. sojae*, 5 mm diameter mycelial plugs (10-day-old) or zoospores (1 × 10^5^/mL, 10 μL droplets) were used for *P. infestans* infection. Infected leaves were then further incubated at 16 °C.

Disease severity was presented visually 2 to 3 days following inoculation and ranked from 0 (no symptoms) to 4 (rotten leaf) as described for inoculation of *Arabidopsis* with *P. sojae*. Subsequent infection of plants with *P. parasitica* were carried out with appropriate controls as Pan et al. [52] described. Assays were conducted by placing droplets of *P. parasitica* spores or mycelial plugs (5-day old, 2 mm diameter) on the abaxial detached leaf surface and incubating for up to 3 days at 100% relative humidity and 23 °C. All infected leaves were divided into four grades according to the pathogen colonization area ratio (Grade 0, <10%; Grade 1, <25%; Grade 2, <50%; Grade 3, >50%). *Agrobacterium*-mediated transient expression in *N. benthamiana* was carried out as described previously [91]. Our constructs were introduced to the *A. tumefaciens* strain GV3101.

After culturing in liquid LB medium at 28 °C, the cells were collected by centrifugation and resuspended in infiltration solution [10 mM 2-(N-morpholine)-ethane sulfonic acid (MES), 10 mM MgCl_2_, 200 μM acetosyringone]. The densities of cell suspension in infiltration solution were adjusted to an OD_600_ of 0.3, then the cells were incubated for 2 h at room temperature, before infiltration into leaves of 5- to 6-week-old *N. benthamiana*. After 24 h, infiltrated *N. benthamiana* leaves were cut and transferred into moist sealed plastic trays and inoculated with different *Phytophthora* at the infiltration sites. At least 8 leaves from different tobaccos were used for each experiment and five independent experiments were performed. Lesion diameters were recorded at 2–5 dpi. For infecting *Arabidopsis* leaves, the non-parametric data were analyzed using the Wilcoxon Rank-Sum test to evaluate the statistical significance of the plant disease severity. If the data were suitable for conducting parametric tests, just like the lesion diameters from *N. benthamiana*, then Student’s paired *t*-test was conducted.

*Phytophthora* spp. colonization of *A. thaliana* and *N. benthamiana* was further quantified using genomic DNA qRT-PCR, where the *P. sojae Actin* gene (*PsACT*, JGI GeneID:108986), *P. infestans O8* gene (*PiO8*), *P. parasitica UBC* gene (*PpUBC*, PPTG_09948), *Arabidopsis UBC9* gene (*AtUBC9*, *At4G27960*), and *N. benthamiana Actin* gene (*NbAct*, Niben101Scf09133g02006.1) were amplified [50,88,92]. Leaf discs were punched out of *A. thaliana* (6 mm diameter) leaves at 6, 24, and 48 hpi with *P. parasitica*. Total genomic DNA was extracted using the CTAB method. *N. benthamiana* (15 mm diameter) leaves were respectively collected at 2, 3, and 5 dpi with *P. parasitica*, *P. sojae*, and *P. infestans*. The experiments were replicated three times, with four discs from four different leaves per biological replicate.

### 4.5. Microscopy

To observe *Phytophthora* invasion, we added suitable zoospores to detached leaves as described above. Specimens were mounted on microscope slides and analyzed with different microscopes. For GFP visualization, confocal laser scanning microscopy images were obtained with an Olympus FV3000 confocal microscope (Olympus, Shinjuku, Japan). Trypan blue stained leaves were visualized under an Olympus BX-51 microscope. Microscopy images captured for the various infections were analyzed using the Olympus FLUOVIEW FV1000, Zeiss ZEN, ImageJ (1.6.0) and Adobe Photoshop CC2019.

### 4.6. Transcriptome Analysis and Gene Expression Assays

Total RNAs isolated from uninfected detached leaves of Col-0, VQ28OE9, and VQ28KO12 were sequenced with an Illumina Novaseq™ 6000 (LC-Bio Technology CO., Ltd., Hangzhou, China). After removing the low-quality and undetermined bases, we used HISAT2 software [93] to map reads to the genome. The mapped reads of each sample were assembled using StringTie [94] with default parameters. Then, all transcriptomes from all samples were merged to reconstruct a comprehensive transcriptome using gffcompare. After the final transcriptome was generated, StringTie and ballgown [95] were used to estimate the expression levels of all transcripts and determine expression levels of mRNAs by calculating FPKM (Fragments Per Kilobase of exon model per Million mapped reads). The differentially expressed mRNAs were selected with fold change >2 or fold change <0.5 and *p* value < 0.05 by R package edgeR [96] or DESeq2 [94], and then Gene Ontology (GO) enrichment and Kyoto Encyclopedia of Genes and Genomes (KEGG) enrichment were conducted for the differentially expressed mRNAs. For *VQ28* gene transcription level analysis, SYBR green qRT-PCR analysis was performed. The 4-week-old *Arabidopsis* leaves were inoculated by *P. sojae* and/or *P. parasitica* at sequential time points within 72 h. Total RNA was extracted using the Trizol reagent (Invitrogen, Carlsbad, CA, USA) and reverse transcribed using PrimeScript™ RT Reagent Kits with gDNA Eraser (TaKaRa, Dalian, China) according to the product manuals. SYBR Green (CWBIO, Taizhou, China) was used as fluorescent reporter dye to amplify the target genes and was normalized to the *A. thaliana EF1A* gene (*AT5G60390*) [97]. After the reaction, 20–50 ng cDNA was subjected to qRT-PCR on a Roche LightCycler 480 Real-time PCR machine. At least three independent biological samples were used for qRT-PCR analysis for every result. Gene-specific primers for qRT-PCR are listed in Table S1.

### 4.7. Phytohormone Measurment

To examine abscisic acid (ABA), jasmonic acid (JA) and salicylic acid (SA), fresh *A. thaliana* leaves were collected, extracted, and quantitated using high performance liquid chromatography-mass spectrometry (HPLC-MS) QTRAP 5500, as previously described [98] with minor modifications. Briefly, 0.1 g sample (three independent biological replicates) was ground into fine powder, and mix with 1 mL extraction solution (methanol:isopropanol = 1:4) by vortexing for 5 min, followed by storage overnight at −20 °C. Then, the samples were centrifuged at 12,000× *g* for 10 min at 4 °C, and the supernatant was transferred to a 1.5-mL centrifuge tube. Approximately 200 μL supernatant were pipetted into a new centrifuge tube and blown-dried with nitrogen. After the mixture of 200 μL sodium acetate buffer (pH = 5.5, 0.1 M) for 1 h at 4 °C, the liquid was centrifugated and 200 μL supernatant was transferred to a new centrifuge, followed by mixing with 10U β-glucosidase for 1.5 h at 37 °C. Following centrifugation at 14,000× *g* for 10 min, the supernatant was collected and ready for analysis. The analytical conditions were as follows: HPLC column, SHIMADZU intertsil OSD-4C18 (3.5 μm, 150 mm × 3.0 mm); mobile phase A, solvent system, water (0.1% methanoic acid): methanol; gradient program, 75:25 *v*/*v* at 0–1 min, 5:95 *v*/*v* at 1.0–5.0 min, 5:95 *v*/*v* at 5.0–6.5 min, 75:25 *v*/*v* at 6.5–6.6 min, and 75:25 *v*/*v* at 6.6–10.0 min; flow rate, 0.3 mL/min; temperature, 25 °C; and injection volume, 5 μL. The effluent was alternatively connected to an ESI-triple quadrupole linear ion trap (QTRAP)-MS. LIT and triple quadrupole (QQQ) scans were acquired by an API 6500 QTRAP LC/MS/MS triple quadrupole-linear ion trap mass spectrometer (QTRAP) system. The ESI source operation parameters were the same as that descripted by Müller and Munné-Bosch [99].

## Data Availability

We have submitted our WGS and RNA-seq data to NCBI and chose the option to keep the data private until manuscript acceptance.

## Supplementary Materials

The following supporting information can be downloaded at: https://www.mdpi.com/article/10.3390/plants11070858/s1, Figure S1: Leaves of *A. thaliana* wild-type Col-0, VQ28 knock-out mutant KO12, and overexpressing line OE9 inoculated by *P. infestans* were taken at 4 dpi under fluorescence or bright light after being stained by trypan blue. Arrows indicate reproductive structures, secondary hyphae (hy) and sporangia (sp). Bars indicates 200 μM in the bigger photos and 50 μM in smaller ones; Figure S2: Overexpression of *VQ28* significantly compromised resistance to *P. infestans* and *P. parasitica*. (A,B) The phenotypes of WT Col-0, two knock-out lines (KO4 and KO12), and three VQ28 overexpression plants (OE2, OE9, and OE10) at 7 d and 60 h after inoculation with *P. infestans* (A) and *P. parasitica* (B), respectively. The experiments were performed at least three times and at least 10 leaves of every line were used per replicate. (C,D) Graphs of ratios for susceptibility levels of the plants infected by *P. infestans* (C) and *P. parasitica* (D). Columns with one or two asterisks show significance under *p* < 0.05 or *p* < 0.01, respectively. (E) Biomass of *P. parasitica* in the infected plant tissues at 6, 24, and 48 hpi was determined by qRT-PCR. Primers specific for *Phytophthora UBC* genes and *A. thaliana UBQ9* gene were used. (F,G) *N. benthamiana* leaves infiltrated with *A. tumefaciens* GV3101 harboring 35S::VQ28CMyc or 35S::MycGFP construct were challenged by *P. infestans* (F) and *P. parasitica* (G), respectively. Data are presented as mean ± SD from at least 8 leaves of four biological replicates in three independent experiments. The data analysis using Student’s *t*-test of lesion diameters shows that overexpressed VQ28 conferred enhanced susceptibility. Asterisks indicate significant differences (* *p* < 0.05, ** *p* < 0.01); Figure S3: Expression analysis of *AT3G03980* by quantitative RT-PCR in *A. thaliana* infected with *P. sojae* and *P. parasitica*. Accumulation of *AT3G03980* transcripts was minimally up-regulated upon *P. parasitica* infection; Figure S4: The *AT3G03980* gene did not mediate plant resistance to *P. parasitica*. (A) Quantitative RT-PCR analysis indicated the mRNA levels of *AT3G03980* in rosette leaves from overexpression transformants (980OE2, 980OE3, and 980OE5) and RNA silenced plants (980KD6, 980KD9, and 980KD16). Data represented the ratio of *AT3G03980* expression between transgenic lines and wild-type Col-0. *EF1A* was used as the internal control. Bars represent standard errors from three biological replicates and asterisks show statistical significance based on Student’s *t*-test (** *p* < 0.01). (B) Statistical analysis of plant disease severity. All infected leaves were divided into four grades (Grade 0, <10%; Grade 1, <25%; Grade 2, <50%; Grade 3, >50%) depending on the area ratio of water-soaked lesions. (C) Leaf inoculation assay showed that *AT3G03980* overexpression and knock-down lines and Col-0 were susceptible to *P. parasitica* with no difference, scored 2 dpi. At least ten leaves from ten different plants were tested in each experiment, and three independent experiments were performed; Figure S5: NPVQ28::GUS plants were stained at different growth stages. (A–D) 1-, 2-, 5- and 11-day-old transformants, respectively. (E) Inflorescence. (F) Rosette leaf from 35-day-old *A. thaliana*. Bars indicate 500 μm in (A,B) and 2 mm in (C–F); Figure S6: Relative expression differences of *AtVQ* family by RNA-seq of leaves and roots upon *P. parasitica* infection. (A) Four-week-old *A. thaliana* wild-type Col-0 leaves inoculated by *P. parasitica* zoospore suspension (2000 spores per leaf) were gathered at 24 and 48 hpi. (B) 2-week-old seedlings were inoculated by dipping the roots into a 100 spores/μL *P. parasitica* zoospore suspension for approximately 5 s, followed by transferring to 1/2MS plates without sugar. The infected roots were collected at 12 and 24 hpi, respectively; Figure S7: *VQ28* was induced by wounding. qRT-PCR detection for the *VQ28* expression in Col-0 leaves at different time points after wounding. (A) The qRT-PCR data showed *VQ28* was up-regulated by wounding. Total RNA of wild-type Col-0 was isolated from leaf samples, and transcript levels were detected by qRT-PCR with *A. thaliana EF1A* gene as internal control. Error bars indicate SD (*n* = 3). (B) β-Glucuronidase (GUS) staining of 30-day-old NPVQ28::GUS rosette leaves 6 h after wounding. Bars indicate 200 μm; Figure S8: Expression levels of ABA-related genes determined by RT-PCR of uninfected Col-0 and VQ28OE9 leaves. Total RNAs were obtained from leaves of Col-0 and VQ28OE9 without infection; Figure S9: The C-terminal ends and VQ-motif of VQ28 were essential in susceptibility. The *N. benthamiana* leaves expressing VQ28Δ1-81::Myc, VQ28ΔVQ::Myc, VQ28Δ82-208::Myc, and MycGFP were infected by *P. parasitica* (A) and *P. infestans* (B). Quantification of lesions in *N. benthamiana* leaves measured at 2 and 5 dpi respectively. Error bars represent the standard deviation (SD) of more than eight leaves, and asterisks denote significant differences from the MycGFP control group (Student’s *t*-test; * *p* < 0.05); Figure S10: Transcriptome analysis of *VQ28* and 14 *WRKY* genes with significantly different expression during the infection of *A. thaliana* leaves by *P. parasitica. A. thaliana* leaves were inoculated with *P. parasitica* and samples were collected at 0, 24, and 48 hpi. Asterisks denote significant differences from the 0 h control group (Student’s *t*-test; ** *p* < 0.01); Table S1: Primers used in this study; Table S2: There were 14 WRKYs were enriched in VQ28OE9 compared with Col-0.
