# Supplementary material for: Activation of the VQ Motif-Containing Protein Gene VQ28 Compromised Nonhost Resistance of Arabidopsis thaliana to Phytophthora Pathogens
Source: Plants (Basel). 2022 Mar 24;11(7):858. doi: 10.3390/plants11070858 (PMC9002740; doi:10.3390/plants11070858)
Supplement: Supplementary file 1 [file plants-11-00858-s001.zip › plants-1642976-supplementary.pdf]

Supplementary Figures and Tables

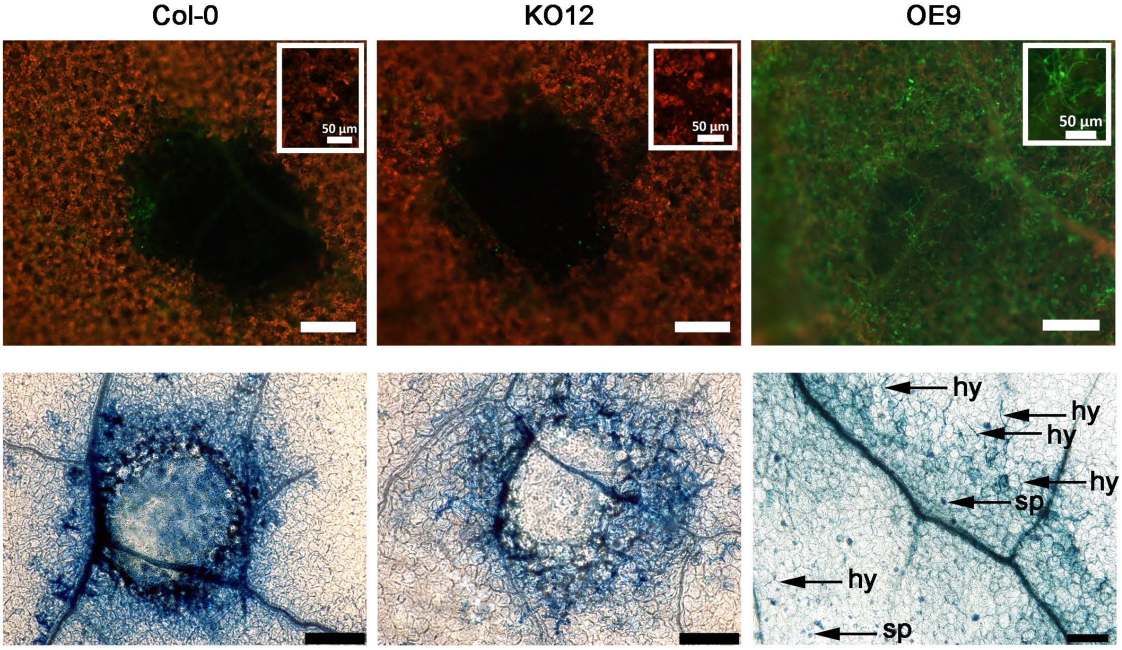

Figure S1. Leaves of *A. thaliana* wild-type Col-0, *VQ28* knock-out mutant KO12, and overexpressing line OE9 inoculated by *P. infestans* were taken at 4 dpi under fluorescence or bright light after being stained by trypan blue. Arrows indicate reproductive structures, secondary hyphae (hy) and sporangia (sp). Bars indicate 200 µM in the bigger photos and 50 µM in the smaller ones.

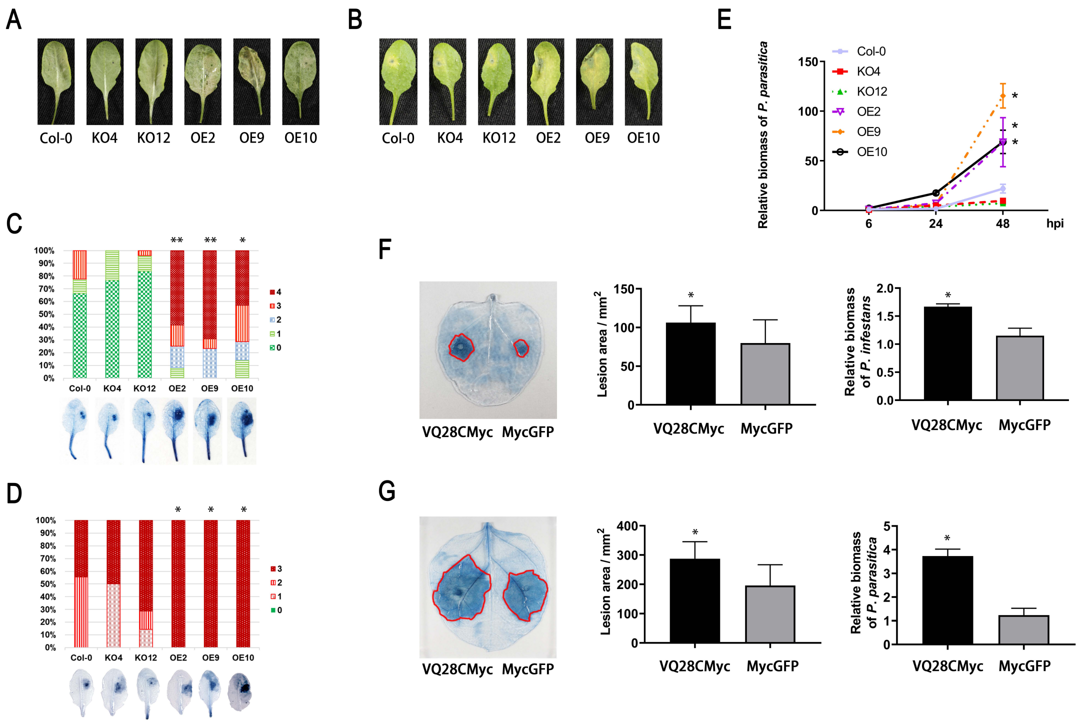

Figure S2. Overexpression of *VQ28* significantly compromised resistance to *P. infestans* and *P. parasitica*. (**A**,**B**) The phenotypes of WT Col-0, two knock-out lines (KO4 and KO12), and three *VQ28* overexpression plants (OE2, OE9, and OE10) at 7 d and 60 h after inoculation with *P. infestans* (**A**) and *P. parasitica* (B), respectively. The experiments were performed at least three times and at least 10 leaves of every line were used per replicate. (**C**,**D**) Graphs of ratios for susceptibility levels of the plants infected by *P. infestans* (**C**) and *P. parasitica* (**D**). Columns with one or two asterisks show significance under $p < 0.05$ or $p < 0.01$, respectively. (**E**) Biomass of *P. parasitica* in the infected plant tissues at 6, 24, and 48 hpi was determined by qRT-PCR. Primers specific for *Phytophthora UBC* genes and the *A. thaliana UBQ9* gene were used as the internal control. (**F**,**G**) *N. benthamiana* leaves infiltrated with *A. tumefaciens* GV3101 harboring 35S::VQ28CMyc or 35S::MycGFP construct were challenged by *P. infestans* (F) and *P. parasitica* (G), respectively. Data are presented as mean ± SD from at least 8 leaves of four biological replicates in three independent experiments. The data analysis using Student's *t*-test of lesion diameters shows that overexpressed VQ28 conferred enhanced susceptibility. Asterisks indicate significant differences (* $p < 0.05$, ** $p < 0.01$);

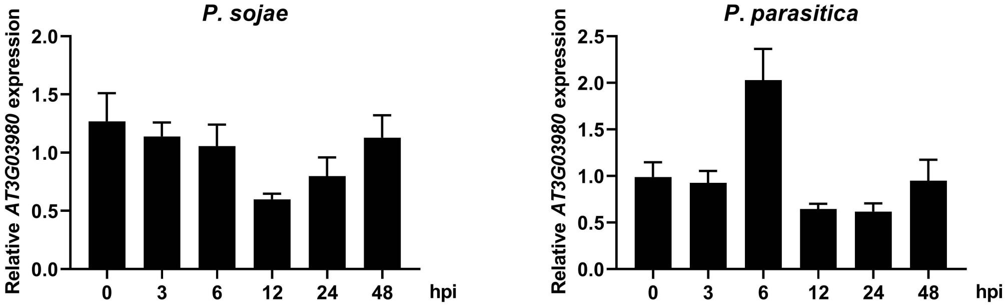

Figure S3. Expression analysis of *AT3G03980* by quantitative RT-PCR in *A. thaliana* infected with *P. sojae* and *P. parasitica*. Accumulation of *AT3G03980* transcripts was minimally up-regulated upon *P. parasitica* infection.

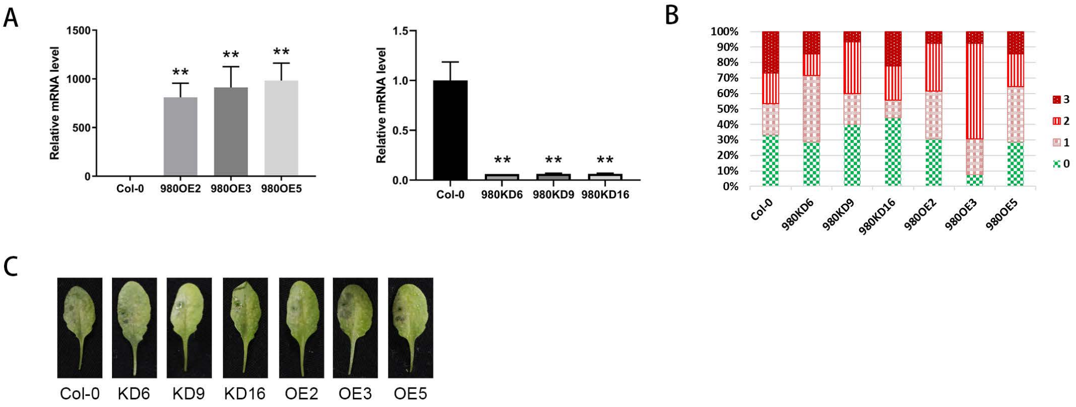

Figure S4. The *AT3G03980* gene did not mediate plant resistance to *P. parasitica*. (**A**) Quantitative RT-PCR analysis indicated the mRNA levels of *AT3G03980* in rosette leaves from overexpression transformants (980OE2, 980OE3, and 980OE5) and RNA silenced plants (980KD6, 980KD9, and 980KD16). Data represented the ratio of *AT3G03980* expression between transgenic lines and wild-type Col-0. *EF1A* was used as the internal control. Bars represent standard errors from three biological replicates and asterisks show statistical significance based on Student's *t*-test (** $p < 0.01$). (**B**) Statistical analysis of plant disease severity. All infected leaves were divided into four grades (Grade 0, <10%; Grade 1, <25%; Grade 2, <50%; Grade 3, >50%) depending on the area ratio of water-soaked lesions. (**C**) Leaf inoculation assay showed that *AT3G03980* overexpression and knock-down lines and Col-0 were susceptible to *P. parasitica* with no difference, scored 2 dpi. At least ten leaves from ten different plants were tested in each experiment, and three independent experiments were performed.

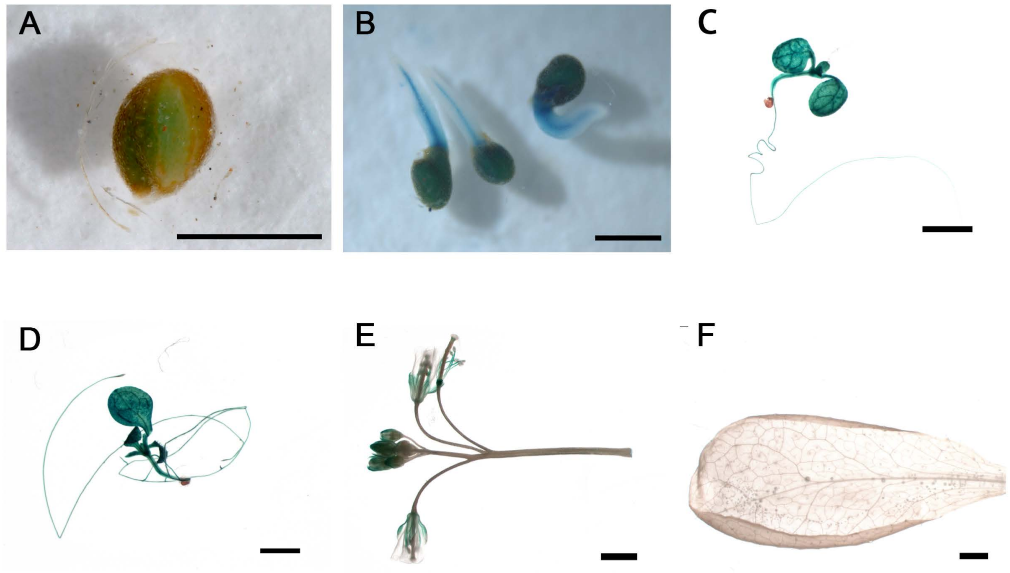

Figure S5. NPVQ28::GUS plants were stained at different growth stages. **(A-D)** 1-, 2-, 5- and 11-day-old transformants, respectively. **(E)** Inflorescence. **(F)** Rosette leaf from 35-day-old *A. thaliana*. Bars indicate 500 μm in **(A**,**B)**, and 2 mm in **(C)** to **(F)**.

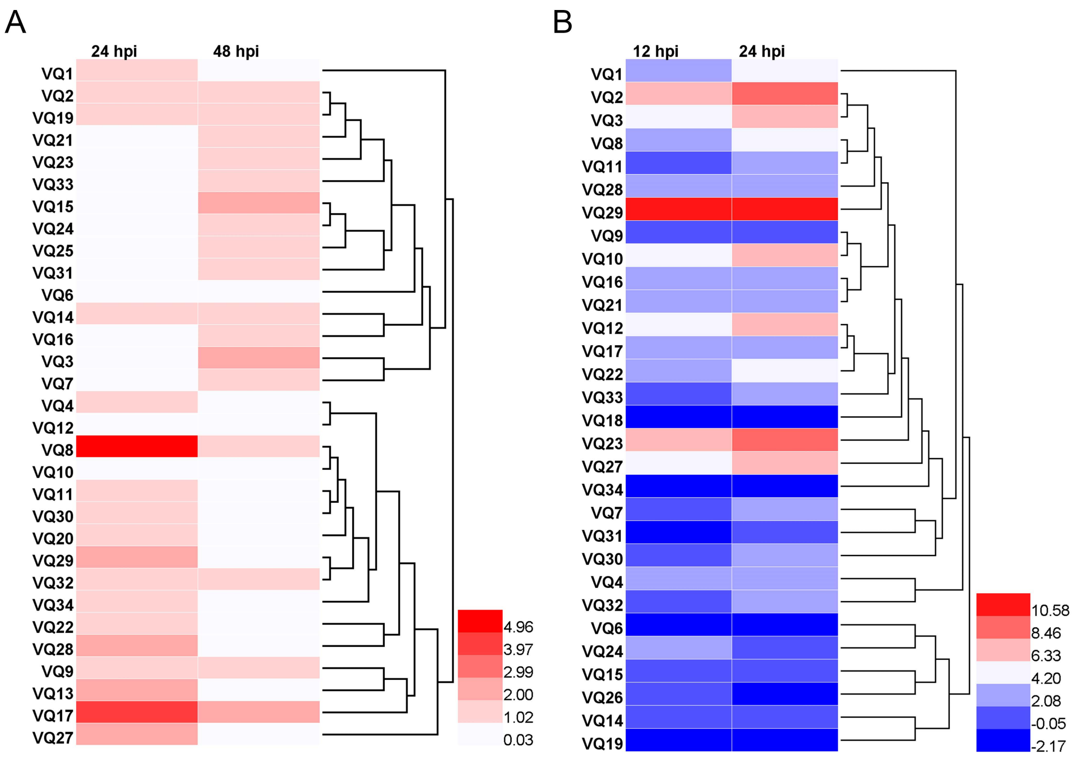

Figure S6. Relative expression differences of *AtVQ* family by RNA-seq of leaves and roots upon *P. parasitica* infection. **(A)** Four-week-old *A. thaliana* wild-type Col-0 leaves inoculated by *P. parasitica* zoospore suspension (2000 spores per leaf) were gathered at 24 and 48 hpi. **(B)** Two-week-old seedlings were inoculated by dipping the roots into a 100 spores/μL *P. parasitica* zoospore suspension for approximately 5 seconds, followed by transferring to 1/2MS plates without sugar. The infected roots were collected at 12 and 24 hpi, respectively.

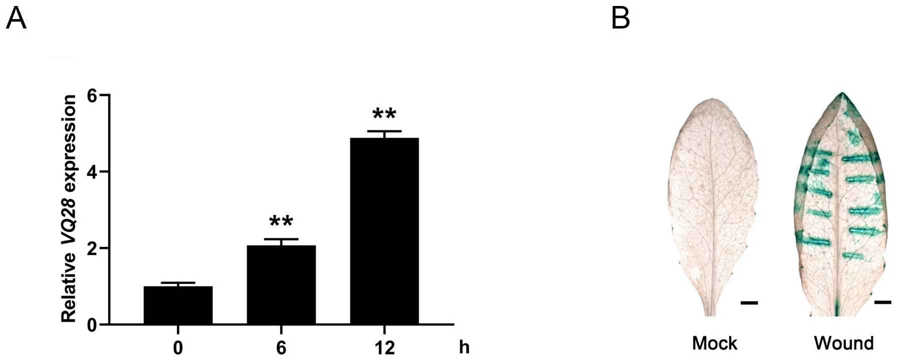

Figure S7. *VQ28* was induced by wounding. qRT-PCR detection for the *VQ28* expression in Col-0 leaves at different time points after wounding. **(A)** The qRT-PCR data showed *VQ28* was up-regulated by wounding. Total RNA of wild-type Col-0 was isolated from leaf samples, and transcript levels were detected by qRT-PCR with the *A. thaliana EF1A* gene as the internal control. Bars represent SD from three biological replicates and asterisks indicate statistical significance based on a Student's *t*-test (** $p < 0.01$). **(B)** β-Glucuronidase (GUS) staining of 30-day-old NPVQ28::GUS rosette leaves 6 h after wounding. Bars indicate 200 μm.

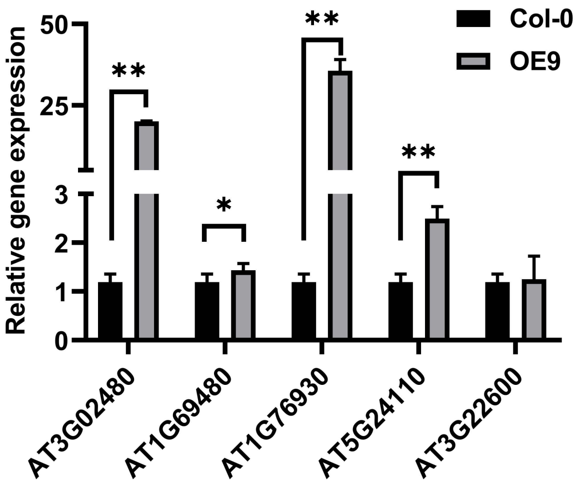

Figure S8. Expression levels of ABA-related genes determined by RT-PCR of uninfected Col-0 and VQ28OE9 leaves. Total RNAs were obtained from leaves of Col-0 and VQ28OE9 without infection. Bars represent SD from three biological replicates and asterisks indicate statistical significance based on a Student's *t*-test (* $p < 0.05$, ** $p < 0.01$).

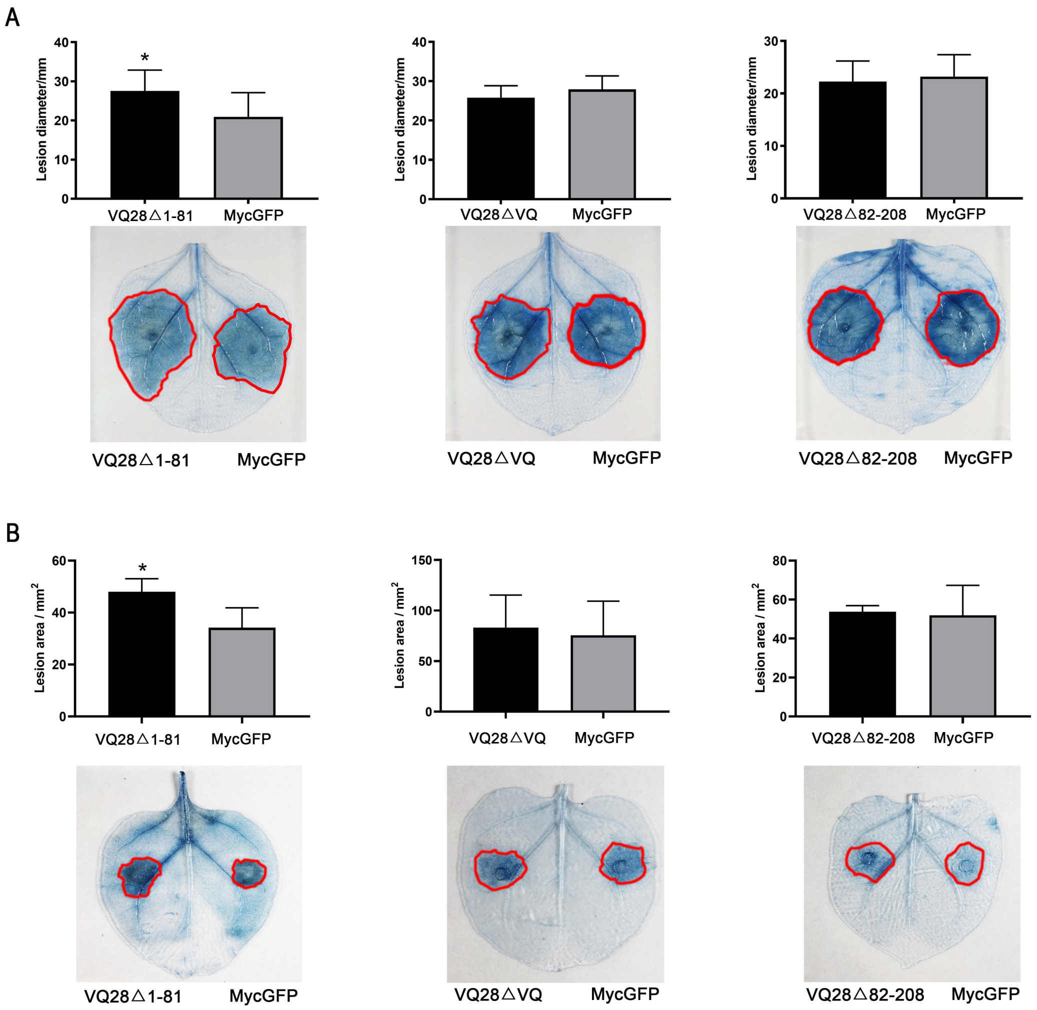

Figure S9. The C-terminal ends and VQ-motif of VQ28 were essential in susceptibility. The *N. benthamiana* leaves expressing VQ28Δ1-81::Myc, VQ28ΔVQ::Myc, VQ28Δ82-208::Myc, and MycGFP were infected by *P. parasitica* **(A)** and *P. infestans* **(B)**. Quantification of lesions in *N. benthamiana* leaves measured at 2 and 5 dpi respectively. Error bars represent the standard deviation (SD) of more than eight leaves, and asterisks denote significant differences from the MycGFP control group (Student's *t*-test; * $p < 0.05$).

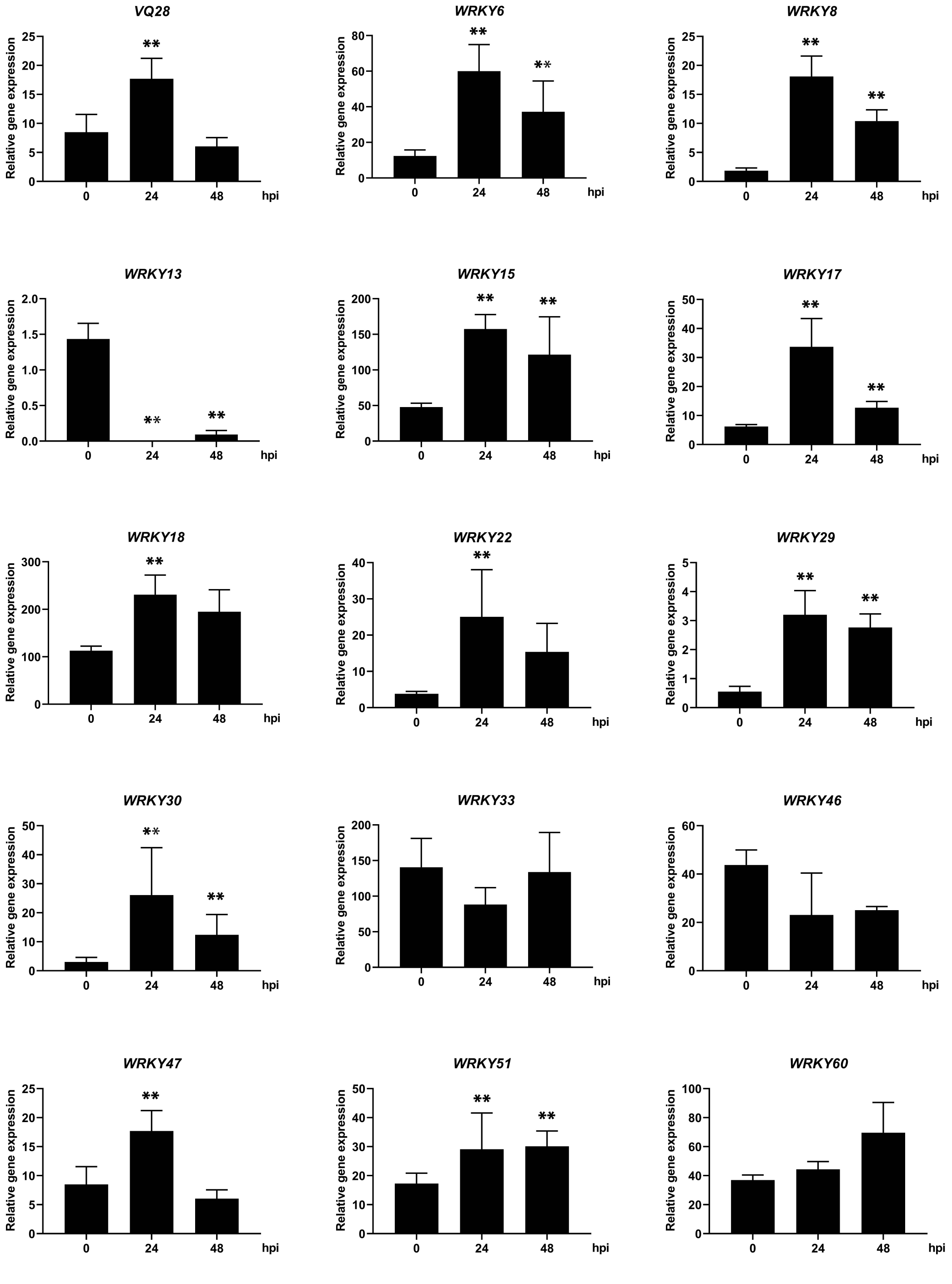

Figure S10. Transcriptome analysis of *VQ28* and 14 *WRKY* genes with significantly different expression during the infection of *A. thaliana* leaves by *P. parasitica*. *A. thaliana* leaves were inoculated with *P. parasitica* and samples were collected at 0, 24, and 48 hpi. Asterisks denote significant differences from the 0 h control group (Student's *t*-test; ** $p < 0.01$).

Table S1. Primers used in this study.

Name	Sequence 5' to 3'
**Flanking sequences around T-DNA insertion sites**	
AT4G27390.1-F	TAGTTCGATTTGTCTATTTTGG
AT4G27390.1-R	AGGAAGTTCATTTCATTTGG
AT3G03890.2-F	CATTTGGAGAGGACACGCTG
AT3G03890.2-R	GCCTTGATGCTGGATTTGGAT
AT4G20010.1-F	GGAGAAGAATCATGGAAGGAC
AT4G20010.1-R	AGAGGACACGCTGAAGCTAG
AT3G03980-F1	CGTCCGCAATGTGTTATTAAGTTG
AT3G03980-R1	CGACCACCACCTCCTTGTTTTA
**Wild type sequences around T-DNA insertion sites**	
AT4G27390WT-F	TTTACCCTTTGCTGTCCTCTA
AT4G27390WT-R	GCTTTGCCCTTTCTGTACTCA
AT3G03890WT-F	AATCCCTCCTTCCTCTCATC
AT3G03890WT-R	TTCCCATACTCTTTTTTGTCG
AT4G20010WT-F	TCATCCTTATCTCACTCGCAA
AT4G20010WT-R	ATCAGGCATTAAAGTACCCCA
AT3G03980WT-F	CAACCGCCGCTTCCTCTC
AT3G03980WT-R	CTTCGCCGACCCTCCCAA
**Knock out of *Arabidopsis VQ28***	
sgR_VQ28-F94	GATTGTCATCGTCGGGATCTTCCCT
sgR _VQ28-R94	AAACAGGGAAGATCCCGACGATGAC
sgR _VQ28-F128	GATTGCGGCACATGAAGCTGCTGGA
sgR _VQ28-R128	AAACTCCAGCAGCTTCATGTGCCGC
**Overexpression of *Arabidopsis VQ28***	
XHOI_VQ28CDS-F	CCGCTCGAGATGAACAACTCTAGAGAAGACC
BamHI_VQ28CDS-R	CGCGGATCCTTATAAATCGAGATCTCTCATCATAA
**Clone full length CDS of *VQ28* in fusion with GFP or Myc**	
NVQ28-F	TTTGGAGAGGACACGCTCGAGATGAACAACTCTAGAGAAGACCAGTGG
NVQ28-R1	CGCCCTTGCTCACCATTAAATCGAGATCTCTCATCATAAACTCTT
CGFP-F1	TTTAATGGTGAGCAAGGGCGAGG
CGFP-R	TCATTAAAGCAGGACTCTAGATTACTTGTACAGCTCGTCCATGCC
NGFP-F1	TTTGGAGAGGACACGCTCGAGATGGTGAGCAAGGGCGAGG
NGFP-R1	TCATCTTGTACAGCTCGTCCATGCC

CVQ28-F1	GGACGAGCTGTACAAGATGAACAACTCTAGAGAAGACCAGTGG
CVQ28-R	TCATTAAAGCAGGACTCTAGATTATAAATCGAGATCTCTCATCATAAACTC
VQ28-CMYC-F	AGAGGACACGCTCGAGATGAACAACTCTAGAG
VQ28-CMYC-R	TTAACCCCATCTCGAGTAAATCGAGATCTCTCATCATAAACT
**Construction of mutanted VQ-motif**	
VQ28ΔVQ-F1	AACCCTAGCAATTTCCGAGCCGAAGACCTGAAATTTACCGGACGTTCTGCCGG
VQ28ΔVQ-R1	CCGGCAGAACGTCCGGTAAATTTCAGGTCTTCGGCTCGGAAATTGCTAGGGTT
VQ28Δ82-208-R	GGACTCTAGAGGATCCTTATCCGGTAAATTTCTGAACTAGGGCTCG
VQ28Δ1-81-F1	AGAGGACACGCTCGAGATGCGTTCTGCCGGAGGTGAATCC
**Overexpression of *Arabidopsis AT3G03980***	
XhoI_980CDS-F	CCGCTCGAGTATGTCTACACATTCATCAATCTCG
XbaI_980CDS-R	GCTCTAGACTTTTATACATATCCACCATTAACAG
**Silencing *Arabidopsis AT3G03980***	
980RNAi1-1F	TTTGGAGAGGACACGCTCGAGGTTAAAATATAAAGTTAGGGGCTGATTTG
980RNAi1-1R	TTATTTCCTTACCAATTGGGGTACCCAATACCACGGGAAGAGC
980RNAi1-2F	GGAAATTGGGTTCGAAATCGATCAATACCACGGGAAGAGC
980RNAi1-2R	TCATTAAAGCAGGACTCTAGAGTTAAAATATAAAGTTAGGGGCTGATTTG
***Arabidopsis* gene as internal control for qRT-PCR**	
UBC9-F	CATCGGATAGCCCTTATTCTG
UBC9-R	TGGAACACCTTCGTCCTAAAA
EF1A-F	TCCAGCTAAGGGTGCC
EF1A-R	GGTGGGTACTCGGAGA
**qRT-PCR test for six ABA-related marker genes**	
L_AT1G69480.1_1950-2249	GTCTATTTTGCAGCCATGGTAG
R_AT1G69480.1_1950-2249	AGCGACTTCAAGTTGAACTCTA
L_AT1G52690_100-399	TCAAGAGTCCAAAGACAAGACA
R_AT1G52690_100-399	GTATATTCAGCTGCATCGTGTG
L_AT3G02480_0-206	CAGACTGGACAACAAATGAAGG

R_AT3G02480_0-206	TTAGTGGCTTTTGTTCATGCC
L_AT1G76930_100-399	TACTCTCCTCCTCCTGTTTACA
R_AT1G76930_100-399	GGAGGAGGGGAGTAGTACTTAA
L_AT3G22600_250-512	AACTCTTCAGGACCAGGTAATG
R_AT3G22600_250-512	GAAGATTGCCATGTAGGAAACC
At5G24110-F	CGCTGGACGATGGATTCAGTTGGAGA
At5G24110-R	TCGGTTCGAGGTTTTGTATCGGCATTG
qNCED3-F1	TCCAGATTGCTTCTGCTTCCAT
qNCED3-R1	GGACCCTATCACGACGACTTCA
qABA1-F	TTGTTTGGCCGTAGTGAAGCT
qABA1-R	AGACTCGATATCCGCTGGTATAAAA
qCYP707A1-F2	AGAGTCTCTAACTTGGGGAGAT
qCYP707A1-R2	TGGATCAAATTTCCCCGGATTA
**qRT-PCR test for six JA-related marker genes**	
qAOC2-F	TCATCGAACACGTCCCAGAG
qAOC2-R	CAAAGATTCCAGCACCACCA
qAOC3-F	GAAGACACGTTCCTCGCTGT
qAOC3-R	CTGTCCGTACGCACCTTCA
qLOX3-F	GACGAGACGAGCAATTTGAA
qLOX3-R	GCTTTGAGCGACGGAATTAG
qLOX4-F	CGAGGGCTTGCTTAGATACG
qLOX4-R	CCGGTGGATAAGACGTGACT
qCYP94B3-F1	CATATTCAATGTCGACGGTCAC
qCYP94B3-R1	AAAAGCAAAACTCCTAAGCGAG
**qRT-PCR test for six SA-related marker genes**	
qPAL1-F	TGTAGCGCAACGTACC
qPAL1-R	GTTCGGGATAGCCGATG
qICS1-F	GCCGTCTCTGAACTCAAATCTCAA
qICS1-R	CTCCAATCGTCATGAGAGGAA
qCBP60g-F	AAGAAGAATTGTCCGAGAGGAG
qCBP60g-R	GGCGAGTTTATGAAGCACAG
**qRT-PCR test for identification the expression of the genes in the vicinity of the four T-DNA insertions in *esp1***	
L_AT3G03900.1_0-299	CAGTGGGAAATTCAACGAACAT
R_AT3G03900.1_0-299	CATGACGAAGATTGTCACCATC
L_AT3G03910.1_400-699	CTGATGTTCCAGCTCCTGATAT
R_AT3G03910.1_400-699	GTTGAGTAAAGCTTCTGTTGCA
L_AT3G03920.1_100-399	TTTAGAGACGAAGGTCCTCCTA
R_AT3G03920.1_100-399	TGGGCCAAAGATTTCATCTACT
L_AT3G03930.1_350-649	ATCCAGCAATCATACAAAACCG
R_AT3G03930.1_350-649	ACTTGGGTTCAAAAAGTCAACC

L_AT3G03940.1_900-1199	CAATAAGGTATGCAAGTTGCCA
R_AT3G03940.1_900-1199	ACACATCAATTCAGGAGAGGTT
L_AT3G03880.1_0-299	GTCTTAGAGCCAAAGCCAAAAA
R_AT3G03880.1_0-299	GGTAGTCCAGATCTGAGGATTG
L_AT3G03870.2_100-399	CTAAAGATCCTCTCCCCAAGAC
R_AT3G03870.2_100-399	ATTGGGTATCTTCGTGTTGTCT
L_AT3G03860.1_200-499	AAATACAACACCTAGCTGTGGA
R_AT3G03860.1_200-499	CCATGGTACCGAGCATTTAAAG
L_AT3G03850_0-281	TAGTAAAAACAAGCAAGGCACC
R_AT3G03850_0-281	GATCTTGAAACAAAGGCTGGTT
qAT3G03840_F2	GAGGCTCATTAGCAGGAATG
qAT3G03840_R2	CGAACTCTTCCTCGGATTTAC
L_AT4G27400.1_401-589	CTCCCTTGAGACCACCAAAG
R_AT4G27400.1_401-589	CGGATGAGTACCAAGTGGATAAA
L_AT4G27410.3_300-599	GCAACGGGTACTGACAAAATTA
R_AT4G27410.3_300-599	GTAAATTCGACACAACACCCAA
L_AT4G27420.1_1100-1399	CTCTTTCCTCTGTGGTCTTCTT
R_AT4G27420.1_1100-1399	TGAGATAAAGAACAACAGCCCT
L_AT4G27430.1_350-649	GTTGTTGGGTTTGAACTTGACT
R_AT4G27430.1_400-699	ATTTGGTCCACCCACATTCTAT
L_AT4G27435.1_0-299	TGTTTAACCTCATTGCCTTTGG
R_AT4G27435.1_0-299	AATGAAGAGAATGAGGGCAAGA
L_AT4G27370.1_1350-1649	CTATGTTAATGGGCTGCAACTC
R_AT4G27370.1_1350-1649	GATGATTTTCGCCAGAGAATCC
qAT4G27360_F2	CTACTCAAATTGCCCGTTTCATC
qAT4G27360_R2	GTTCACCGACTGATCCCTTAAA
qAT4G27350_F2	GAGACGCTGCTCAAATCTATC
qAT4G27350_R2	CGTCTCCGTCTACTTTCATTC
L_AT4G27340.1_1450-1749	AGTGTTGTCAATAAGCTCGAGA
R_AT4G27340.1_1450-1749	TATCGTTGTATACTCCGCGAAA
L_AT4G27330.1_600-899	AGCTTCAGTTTCTGCTTCTAGT
R_AT4G27330.1_600-899	TCCATTTCTAGGGTTTCCTTCC
L_AT4G19970.1_550-849	TACTTGAAACTTATTTGGCGGC
R_AT4G19970.1_550-849	GGTCTTCCATTGTAATCATCGC
qAT4G19975_F2	TGTTAACAATCTCAGCTCTCGT
qAT4G19975_R2	TGTCCGATACGAAAACTCTCAA
L_AT4G19980.1_0-299	GTGACAAAGAAGTCCAATGTCC
R_AT4G19980.1_0-299	CGCAAGTAGTAGTCCTCATCAT
qAT4G19985_F2	GCTACTCACAAGCAGAGACATT
qAT4G19985_R2	CCCATCCTGTCATCTCCAAAC
L_AT4G19990.2_1800-2099	ATCGGTTACAGCTCAAGATCTT
R_AT4G19990.2_1800-2099	TCTTGCAAAGCAGTTACTTTCC
L_AT4G20000.1_100-399	CTAAACGCAAACCCTAGCAATT
R_AT4G20000.1_100-399	TTTGGAGATTGTAGTGGGAGAC

L_AT4G20020.1_200-499	TCGCTGCTTATGAAGAAACTTG
R_AT4G20020.1_200-499	AATCTGGCAAGATGAAAACCAC
L_AT4G20030.1_50-349	ACAGGAACCTTAGGTTTCCAAA
R_AT4G20030.1_50-349	GGCAAGAAAAGCATCATCTTGA
AT4G20040.1_950-1249	TAGTCATAGAAGACCCTGTCCA
R_AT4G20040.1_950-1249	TGTTGTTGACGATGTTTACACC
L_AT4G20050.1_0-299	TAAGGAAATCTCAAGTGGCCAT
R_AT4G20050.1_0-299	GGATCGGCACCATATGATATCA
L_AT4G20060.1_400-699	TTTCAGCAGTATGGTTTCTCCT
R_AT4G20060.1_400-699	GTTTAACATCCCCAGAACAACC
L_AT3G03930.1_0-299	ATTCATGCCCTGAGAAGAAAGA
R_AT3G03930.1_0-299	GCAATGTTGTGGTGAAATCTCT
L_AT3G03940.1_900-1199	CAATAAGGTATGCAAGTTGCCA
R_AT3G03940.1_900-1199	ACACATCAATTCAGGAGAGGTT
L_AT3G03950.3_750-1049	AAAGAAAACTCCCAAGAATGCC
R_AT3G03950.3_750-1049	CAGGGAAACAACCAATCCATTT
L_AT3G03960.1_200-499	TGAACTTCTTCAAAACGCTGAG
R_AT3G03960.1_200-499	TTACGTACATCCATGGTCTCAG
L_AT3G03970.1_550-849	CACTTGTTATGGCACTTTCCAA
R_AT3G03970.1_550-849	GAATCTCCCTCAACAACACCTA
L_AT3G03980.1_450-749	GGTCGGATCATACTTCTGACAT
R_AT3G03980.1_450-749	CATCAAAGAACATCTCAGTGGC
L_AT3G03990.1_350-649	ACTTTGTTTAATATGCGTCCGG
R_AT3G03990.1_350-649	GTCGGAACTCTAACTAACCCAA
L_AT3G04000.1_0-299	TCGAGTAGCTATAGTCACAGGA
R_AT3G04000.1_0-299	ATATCAGCCTTCACCACAATGA
qAT3G04010_F2	ACCGAAATTCCCGGTAGA
qAT3G04010_R2	CCACGACCATTAGGATTAAACA
L_AT3G04020.1_100-399	GAAAGGGTTGCTTTTACTGGAG
R_AT3G04020.1_100-399	CTCTGACTGTATAGCTTCCTCG
L_AT3G04030.3_900-1184	CAGAAGGTCTAAGAGGGAATCC
R_AT3G04030.3_900-1184	GTGTGTTGAGATCTAGCTCTGT

Table S2. There were 14 *WRKYs* were enriched in VQ28OE9 compared with Col-0.

gene_id	Description	FPKM.WT									pval	qval	significant
		0h_1	0h_2	0h_3	24h_1	24h_2	24h_3	48h_1	48h_2	48h_3			
AT4G39410	WRKY13	1.42	1.22	1.66	0	0	0	0.15	0.09	0.04	0.00	0.00	yes
AT2G23320	WRKY15	43.60	53.92	45.91	148.91	142.71	180.57	181.39	103.08	79.72	0.00	0.00	yes
AT2G24570	WRKY17	5.99	5.72	6.99	33.89	23.81	43.33	14.23	13.59	10.21	0.00	0.00	yes
AT4G31800	WRKY18	119.59	116.77	101.89	245.73	184.28	262.48	218.79	224.30	141.41	0.00	0.00	yes
AT5G24110	WRKY30	1.30	4.34	3.45	20.56	13.21	44.48	19.52	12.15	5.49	0.00	0.00	yes
AT2G38470	WRKY33	116.60	187.32	117.85	84.75	66.58	113.43	196.27	115.43	89.63	0.04	0.06	yes
AT2G46400	WRKY46	44.86	49.36	37.12	16.22	10.15	42.82	24.14	26.83	24.26	0.02	0.02	yes
AT5G64810	WRKY51	18.01	20.43	13.31	24.50	19.53	43.25	24.03	33.53	32.77	0.01	0.01	yes
AT2G25000	WRKY60	40.50	36.86	33.39	43.09	50.25	39.77	45.67	83.90	79.28	0.00	0.00	yes
AT5G46350	WRKY8	1.28	2.19	2.04	16.76	15.40	22.09	10.44	12.32	8.47	0.00	0.00	yes
AT1G62300	WRKY6	9.04	15.81	12.30	57.84	46.21	75.96	54.97	36.22	20.57	0.00	0.00	yes
AT4G23550	WRKY29	0.38	0.74	0.54	2.88	2.58	4.15	2.32	3.25	2.72	0.00	0.00	yes
AT4G01250	WRKY22	3.37	4.59	3.49	19.89	15.42	39.83	24.46	11.06	10.67	0.00	0.00	yes
AT4G01720	WRKY47	5.37	8.60	11.49	18.05	14.03	21.04	7.54	6.05	4.51	0.00	0.00	yes
